# Rhein reduces conjugation of IncFII-type plasmids in *Escherichia coli* and mitigates the spread of antibiotic resistance genes

**DOI:** 10.1128/spectrum.03399-25

**Published:** 2026-07-06

**Authors:** Nannan Wang, Haitao Ma, Hanyan Cheng, Xu Yang, Long Liu, Dekang Zhu, Shaqiu Zhang, Xun Zhou, Renyong Jia, Yuanfeng Zou, Lixia Li, Xu Song, Zhongqiong Yin

**Affiliations:** 1 Natural Medicine Research Center, College of Veterinary Medicine, Sichuan Agricultural University 506176, Chengdu, China; 2 Key Laboratory of Animal Disease and Human Health of Sichuan Province, Sichuan Agricultural University 506176, Chengdu, China; Guizhou Medical University, Guiyang, China

**Keywords:** Rhein, IncFII plasmid, Biofilm, horizontal gene transfer, *mcr-1*

## Abstract

Antibiotic resistance genes (ARGs) can be rapidly disseminated via bacterial conjugation, resulting in a substantial decline in the clinical efficacy of antibiotics. Novel therapeutic strategies independent of conventional antimicrobials are urgently needed. In this study, we identified rhein (RHE), a natural anthraquinone compound, as an effective IncFII plasmid transfer-reducing agent. At sub-inhibitory concentrations, RHE significantly reduced the conjugative transfer of IncFII plasmids in *Escherichia coli*. Importantly, the inhibitory effect of RHE extended to clinically relevant contexts, where it markedly impeded the transfer of the *mcr-1* gene among clinical isolates and reduced plasmid dissemination in multiple organs in mouse models, demonstrating its *in vivo* potential. Mechanistic investigations revealed a dual mode of action that distinguishes RHE from traditional antimicrobials. Specifically, RHE compromises bacterial membrane integrity, leading to dissipation of the proton motive force and depletion of intracellular ATP, and concurrently disrupts the bacterial quorum-sensing system. Collectively, these findings establish RHE as a promising lead compound for the development of non-antibiotic therapeutics aimed at limiting the environmental and clinical spread of antimicrobial resistance. This study provides a novel and feasible strategy to address the escalating crisis of ARG transmission.

**IMPORTANCE:**

Antimicrobial resistance in bacteria has become an increasingly severe global health challenge. The widespread dissemination of colistin resistance genes has markedly compromised the clinical efficacy of colistin, underscoring an urgent need for novel strategies to limit the spread of resistance determinants. In this study, we investigated the regulatory effects of rhein on IncFII-type plasmids and evaluated its intervention potential in the transmission of the colistin resistance gene *mcr-1*. Our results demonstrate that RHE effectively reduces *mcr-1* transfer in both *in vitro* and *in vivo* models, highlighting its potential as a promising therapeutic candidate for the prevention and control of antimicrobial resistance gene dissemination.

## INTRODUCTION

Antibiotic resistance genes (ARGs) have become a serious global public health threat. They severely impair clinical treatment outcomes and are widely regarded as emerging environmental contaminants. Horizontal gene transfer (HGT) is a major driver, and plasmid-mediated conjugation is the most efficient route for ARG spread (1). Conjugation plays a pivotal role in the rapid spread of antimicrobial resistance, particularly in dense and nutrient-rich microbial communities such as biofilms and actively growing bacterial populations. In such environments, conjugative plasmids facilitate efficient intercellular ARG transmission. Accordingly, conjugation serves as a core pathway for resistance spread, particularly for plasmid-carried resistance genes in clinical and high-density microbial niches (2, 3).

Among conjugative plasmids, IncFII-type plasmids pose a substantial clinical threat. These plasmids are highly prevalent in Enterobacteriaceae, a family of Gram-negative pathogens responsible for a majority of hospital-acquired infections, and frequently carry multiple ARGs (4, 5). IncFII plasmids are highly prevalent and often carry a “multidrug resistance cassette” encoding genes such as *bla*_CTX-M-55_ gene and *floR* (6, 7). Colistin, an important antibiotic for treating carbapenem-resistant infections, is becoming increasingly ineffective due to the spread of the *mcr* genes. Moreover, the co-transfer of *mcr-1* with *bla*_CTX-M-55_ via IncFII and IncHI2 plasmids or chromosomal integration has driven the persistent dissemination of extensively drug-resistant (XDR) *E. coli* across multiple countries (8). This underscores the urgent need to suppress IncFII plasmid-mediated conjugation to curb the spread of AR.

Conventional strategies for addressing antimicrobial resistance (AMR), including the iterative development of novel antimicrobial agents, have become increasingly unsustainable in the face of the rapid evolutionary diversification of bacterial resistance mechanisms. Blocking the conjugative horizontal transfer of ARGs is a promising and evolutionarily friendly intervention strategy. It can limit the transmission of resistance genes with minimal selective pressure on bacteria, thereby reducing the risk of new AMR generation (9). Previous studies have identified agents capable of curing or suppressing resistance plasmids, including anionic/cationic surfactants, ethidium bromide, and novobiocin (10). However, the inherent toxicity of these compounds greatly limits their application as anti-plasmid agents. For instance, ethidium bromide has mutagenic effects, while cationic surfactants exhibit strong cytotoxicity to mammalian cells (9). Natural compounds remain a crucial source of novel anti-plasmid drugs, owing to their diverse biological activities and the low propensity of bacteria to develop resistance against them. For example, linoleic acid inhibits DNA transfer by suppressing plasmid-encoded TrwD ATPase (9, 11). Thus, there is an urgent need for low-toxicity, multi-target natural inhibitors with anti-conjugative activity, as multi-target mechanisms minimize the risk of resistance evolution.

Rhein (RHE, 5′-dihydroxyanthraquinone-2-carboxylic acid) is a natural anthraquinone compound extracted from medicinal plants such as *Rheum palmatum*, *Cassia obtusifolia*, *Polygonum multiflorum,* and *Aloe vera*. It has become a promising bioactive molecule for pharmacological research (12). Extensive research has corroborated its potent hepatoprotective, neuroprotective, anticancer, and anti-inflammatory bioactivities (13). In addition to the above biological functions, accumulating evidence has confirmed the broad-spectrum antibacterial activity of RHE against both Gram-positive and Gram-negative bacteria, including *Bacillus megaterium*, *Staphylococcus aureus*, and *Cutibacterium acnes* (12). Notably, the antimicrobial activity of RHE has attracted growing research attention in recent years (14). Our previous work demonstrated that RHE restores colistin susceptibility in *mcr-1*-positive strains (15). It is worth noting that existing studies have demonstrated that RHE disrupts bacterial membrane integrity and quorum-sensing (QS) pathways, both of which play a critical facilitative role in mediating bacterial plasmid conjugation (15–17). Conjugation relies on QS-mediated biofilm formation to enhance cell-cell contact, as well as intact membranes and sufficient energy to power T4SS machinery (18, 19). Nevertheless, it remains unclear whether RHE inhibits IncFII plasmid conjugation via the above mechanisms, and its *in vivo* efficacy still lacks systematic verification.

Therefore, this study was designed to evaluate the effect of RHE on IncFII plasmid-mediated ARG transfer *in vitro* and in a murine infection model. We also explored its potential mechanisms by detecting the changes in bacterial membrane integrity, energy metabolism, QS response, and the expression of core conjugation-related genes. Our findings provide a mechanistic basis for the development of RHE as a potential therapeutic agent to combat the spread of antibiotic resistance.

## MATERIALS AND METHODS

### Bacterial strains and reagents

*E. coli* RCAD 0514 and *E. coli* BH126 (IncFII plasmid and *mcr-1*-positive), *Vibrio harveyi* BB170, *E. coli* J53, and *E. coli* DH5α carrying an IncFII plasmid (GenBank: CP034108.1) were kindly provided by the Department of Preventive Veterinary Medicine, Sichuan Agricultural University. Luria-Bertani (LB) broth/agar and Mueller-Hinton (MH) broth were purchased from Qingdao Haibo Biotechnology Co., Ltd. (Qingdao, China). RHE (≥98% purity) was obtained from Chengdu Herbpurify Co., Ltd. (Chengdu, China). Colistin, o-nitrophenyl-β-D-galactopyranoside (ONPG), and 3,3′-dipropylthiadicarbocyanine iodide (DiSC₃(5)) were sourced from Beijing Solarbio Science & Technology Co., Ltd. (Beijing, China). Reactive oxygen species (ROS) detection kit and adenosine triphosphate (ATP) assay kit were purchased from Beyotime Biotechnology Co., Ltd. (Shanghai, China). ExonScript RT SuperMix and UltraStart SYBR Green qPCR Mix were purchased from Rongwei Gene Biotechnology Co., Ltd (Chengdu, China). Female 6–8-week-old SPF ICR mice were purchased from Dashuo Experimental Animal Co., Ltd. (Chengdu, China).

### Growth kinetics determination

Our previous study confirmed that the minimum inhibitory concentration (MIC) of RHE against *E. coli* was 1280 μg/mL (15). To exclude the interference of RHE on bacterial growth, the growth curves of *E. coli* were determined at a series of sub-inhibitory concentrations. Briefly, overnight cultures of *E. coli* DH5α harboring the IncFII plasmid and *E. coli* J53 were diluted 1:1,000 in LB broth. The bacterial suspensions were incubated at 37°C with shaking (200 rpm) in the presence of RHE (0, 40, 80, and 160 µg/mL). Bacterial growth was monitored spectrophotometrically (Thermo Fisher Scientific, USA) by measuring the optical density at 600 nm (OD₆₀₀) every 2 h, and growth curves were plotted accordingly.

### Conjugation experiments

Conjugation experiments were performed using a broth mating protocol as previously described, with minor modifications (20). Briefly, overnight cultures of the donor (*E. coli* DH5α/IncFII plasmid) and recipient (*E. coli* J53) strains were centrifuged (3,000 × *g*, 5 min, 4°C), washed three times with phosphate-buffered saline (PBS), and resuspended in LB broth to a final concentration of ~1 × 10⁸ CFU/mL. Donor and recipient suspensions were mixed at a 1:1 ratio and incubated statically at 37°C for 6, 12, or 24 h in the presence of RHE (40, 80, or 160 μg/mL). LB broth without RHE served as the negative control.

After incubation, bacterial aliquots were serially diluted and plated on selective media: transconjugants were enumerated on LB agar supplemented with ampicillin (Amp; 100 μg/mL) and sodium azide (NaN₃; 100 μg/mL). Total recipients were counted on LB agar containing NaN₃ (100 μg/mL). Spontaneous mutation rates were monitored by plating donor cells on LB agar with Amp (100 μg/mL) and recipient cells on LB agar with NaN₃ (100 μg/mL). All plates were incubated at 37°C for 18–24 h. Transconjugants were confirmed via PCR amplification of the *bla*_CTX-M-55_ gene (Table S1). Conjugation frequency was calculated as the number of transconjugants per recipient. All experiments were performed with three biological replicates.

### *In vivo* conjugation assay

All mice were housed in the Animal Center of the College of Veterinary Medicine, Sichuan Agricultural University, under standard conditions with free access to food and water. The light was from 8:00 am to 8:00 pm, with the temperature kept at 22°C ± 1°C and humidity at 40%–70%. All experimental procedures involving animals were in accordance with the guidelines of the Animal Care and Use Committee of Sichuan Agricultural University. All animal experiments were performed independently of each other with different cohorts of mice.

A murine intraperitoneal infection model was used to evaluate the *in vivo* efficacy of RHE (20). Briefly, 6–8-week-old mice (SPF-grade ICR, female, *n* = 6 per group), weighing approximately 20 g, were intraperitoneally infected with a 1:1 mixture of *E. coli* RCAD 0514 (donor) and *E. coli* J53 (recipient) at a total dose of 2.0 × 10⁸ CFU. Fifteen minutes post-infection, mice received an intraperitoneal injection of 200 µL PBS (control) or RHE (2.5, 5, or 10 mg/kg). At 24 h post-infection, mice were anesthetized via intraperitoneal injection of 300 µL of 1.5% Avertin solution, followed by collection of target organs. The liver, spleen, and kidneys were aseptically excised, homogenized in PBS, serially diluted, and plated onto selective agar media to quantify transconjugant and recipient bacterial loads (CFU/organ). Transconjugants were enumerated on LB agar supplemented with colistin (COL, 10 µg/mL) plus NaN₃ (100 µg/mL). Recipients were counted on LB agar supplemented with NaN₃ (100 µg/mL).

### Membrane permeability assay

Membrane permeability was assessed via ONPG hydrolysis (21). Bacterial suspensions adjusted to OD₆₀₀ = 0.5 were treated with RHE (0, 40, 80, or 160 μg/mL) at 37°C for 1 h. A 100 μL aliquot of the supernatant was transferred to a microplate well containing preheated 3 mM ONPG solution. Following 30 min of incubation at 37°C, o-nitrophenol release was quantified by measuring absorbance at 420 nm.

### Intracellular ATP quantification

Intracellular ATP levels were determined using an ATP assay kit (22). Bacterial cells (OD₆₀₀ = 0.5) were washed with PBS (pH 7.4), exposed to RHE (0, 40, 80, or 160 μg/mL) at 37°C for 1 h, and collected by centrifugation. The cell lysate was mixed with assay reagents to detect supernatant ATP, and luminescence was measured using a multi-mode microplate reader after 5 min of incubation at room temperature.

### Ethidium bromide accumulation assay

The ethidium bromide (EtBr) accumulation experiment was conducted according to previous studies (23). Bacterial cells treated with RHE at concentrations of 0, 40, 80, or 160 μg/mL were incubated at 37°C for 30 min, then harvested, washed, and resuspended in MH broth. Ethidium bromide (EtBr) was added to a final concentration of 4 µg/mL, and the fluorescence intensity was measured after 5 min to assess the intracellular retention of EtBr.

### ROS measurement

Total ROS levels were assessed using the ROS-sensitive fluorescent probe 2′,7′-dichlorodihydrofluorescein diacetate (DCFH-DA). Bacterial suspensions were incubated with 10 μM DCFH-DA at 37°C for 30 min, washed three times with PBS, and then treated with RHE (0, 40, 80, or 160 μg/mL). After 30 min, fluorescence intensity was measured using a microplate reader.

### Membrane depolarization assay

Membrane potential changes were evaluated using the fluorescent dye DiSC₃(5) (0.5 μM) (22). Bacterial suspensions (OD_600_ ≈ 0.5) were washed with PBS, loaded with DiSC₃(5) at 37°C for 15 min, dispensed into a 96-well plate (190 μL/well), and mixed with 10 μL of RHE (0, 40, 80, or 160 μg/mL). Fluorescence intensity was monitored at 5-min intervals.

### RT-qPCR analysis

Donor cells in the early log phase were exposed to sub-MIC levels of RHE (0, 40, 80, or 160 μg/mL) for 8 h and then harvested. Total RNA was extracted using TRIzol reagent, treated with DNase I to remove genomic DNA, and reverse-transcribed into cDNA using ExonScript RT SuperMix. RT-qPCR was performed with UltraStart SYBR Green qPCR Mix. Relative gene expression was calculated using the 2^^(−ΔΔCT)^ method with 16S rRNA as the endogenous control (24). Primer sequences are provided in Table S2.

### Molecular docking analysis

The LuxS protein structure was modeled via homology modeling using *Salmonella* LuxS (PDB ID: 5E68) as the template. His54, His58, Cys128, and Zn^2+^ serve as the active center (25). The three-dimensional structure of RHE was retrieved from the ZINC database. Both the protein and ligand structures were prepared for docking by removing water molecules, adding hydrogen atoms, optimizing protonation states, and performing energy minimization. The prepared LuxS protein model and RHE ligand files were submitted to the AutoDock 4.2 server for molecular docking analysis using standard parameters (26).

### AI-2 bioluminescence assay

The production of the QS signal molecule AI-2 was quantified using the *V. harveyi* BB170 reporter assay (27). Briefly, overnight *E. coli* cultures in LB broth were subcultured (1:100) into fresh LB containing sub-minimum inhibitory concentration (sub-MIC) levels of RHE (0–160 µg/mL). After incubation, the cultures were centrifuged (12,000 × *g*, 10 min, 4°C). Supernatants were filtered (0.22 μm), and stored at −80°C. LB broth served as the negative control. *V. harveyi* BB170 was cultured overnight in 2216E medium at 30°C, diluted 1:5,000, and mixed with filtered supernatant. After 12 h of incubation at 30°C, bioluminescence was measured using a multi-mode microplate reader. AI-2 activity was expressed as relative luminescence units (RLU): (sample luminescence/positive control luminescence) × 100%.

### Biofilm inhibition assay

Biofilm formation was assessed in 96-well microtiter plates. Donor (*E. coli* DH5α/IncFII plasmid) and recipient (*E. coli* J53) strains in the exponential phase were standardized to OD₆₀₀ = 0.5, diluted 1:100 in LB broth, and aliquoted (100 μL/well) into plates containing RHE (40, 80, or 160 μg/mL). After 24 h of static incubation at 37°C, wells were gently washed with PBS, air-dried, and stained with 0.1% crystal violet for 15 min. Bound dye was solubilized in 30% acetic acid, and biofilm biomass was quantified by measuring absorbance at 595 nm (OD₅₉₅).

### Scanning electron microscopy (SEM)

*E. coli* samples were treated with RHE (0 and 160 μg/mL) for 4 h prior to fixation in 2.5% glutaraldehyde at 4°C for 24 h. Fixed cells underwent dehydration through a graded ethanol series (30%, 50%, 70%, 90%, and 100%), followed by critical point drying and sputter-coating with gold-palladium. Morphological changes were visualized using a scanning electron microscope (JSM-IT700HR).

### Statistical analysis

All experiments were performed with at least three biological replicates. Data are presented as the mean ± standard deviation (SD). Statistical analyses were performed using GraphPad Prism 8.3.0. Comparisons between two groups were made using an unpaired two-tailed Student’s *t*-test. For multiple group comparisons, one-way analysis of variance (ANOVA) followed by Dunnett’s multiple comparisons test was used. A *P* value less than 0.05 was regarded as statistically significant. The significance levels were marked with different numbers of asterisks: *P < 0.05, **P < 0.01, ***P < 0.001, and ****P < 0.0001.

## RESULTS

### Rhein prevents IncFII plasmid conjugation *in vitro* at sub-inhibitory concentrations

**Fig 1:**
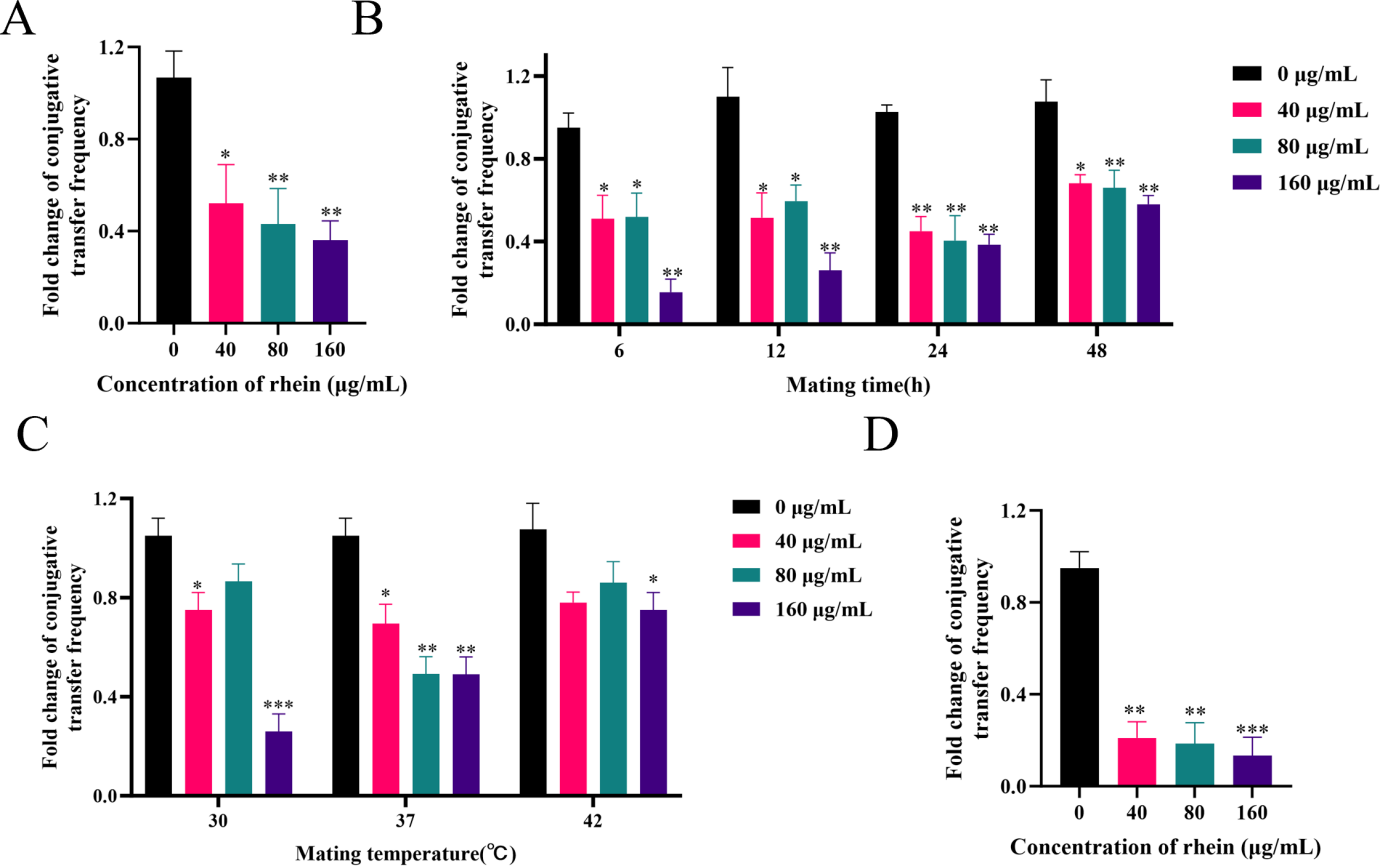
Rhein prevents the transfer of IncFII conjugative plasmid (**A**) and reduces plasmid transfer under different mating times (**B**) and mating temperatures (**C**), along with its capacity to attenuate the dissemination of the *mcr-1* gene (**D**). *P < 0.05, **P < 0.01, ***P < 0.001.

To assess the impact of RHE on the transfer of IncFII plasmids, a conjugation model was constructed using *E. coli* DH5α as the donor strain (carrying the IncFII plasmid) and *E. coli* J53 as the recipient strain. The findings indicated that RHE could reduce plasmid spread, with inhibition rates reaching 48%, 57%, and 64% at concentrations of 40, 80, and 160 μg/mL, respectively (Fig. 1A; Table S4). To rule out the possibility that the effect of RHE stemmed from inhibiting bacterial growth, minimum inhibitory concentration (MIC) analysis and growth kinetics assays were performed. The results showed that the MIC of RHE against the test strains alone was 1280 μg/mL, while its MIC in combination with colistin was 40 μg/mL. Consequently, sub-inhibitory concentrations (0, 40, 80, and 160 μg/mL) of RHE were employed in subsequent experiments. Additionally, bacterial growth curves confirmed that all tested concentrations of RHE had no adverse effects on the normal growth of the strains (Fig. S1). These results confirmed that RHE had no adverse impacts on the growth of donor and recipient strains at all tested concentrations, suggesting that its efficacy in reducing IncFII plasmid transfer was not mediated by the inhibition of bacterial proliferation.

Previous studies have highlighted that conjugation conditions influence transfer frequency, and in the external environment, numerous uncontrollable factors play a critical role in the spread of ARGs. To explore the inhibitory effect of RHE on conjugative transfer under varying conditions, mating time and temperature were selected as variables. Using sub-inhibitory concentrations of RHE, the intraspecific transfer of the IncFII plasmid was investigated, and the corresponding conjugation frequencies were calculated based on our prior methods. This *in vitro* conjugation model confirmed that RHE inhibited the conjugative transfer of IncFII plasmids in *E. coli*, with such inhibition observed across all tested time points and temperatures (Fig. 1B and C; Tables S5 and S6). Meanwhile, MIC determination and PCR assays were used to verify transconjugants. As shown in Fig. S2 and Table S3, both MIC results and agarose gel electrophoresis confirmed that transconjugants carried the IncFII plasmid from the donor strain, validating the suitability of this mating system for further experiments. Given that RHE can restore colistin sensitivity in wild-type strains, we further assessed its impact on the conjugative transfer of *mcr-1*-bearing plasmids in clinical isolates (*E. coli* RCAD 0514 and BH126). Conjugation assays revealed that RHE significantly inhibited the dissemination of colistin resistance genes (*mcr-1*), reducing transfer frequency by 79%–86% (Fig. 1D; Table S7).

### Rhein decreases plasmid dissemination in a murine intraperitoneal infection model

**Fig 2:**
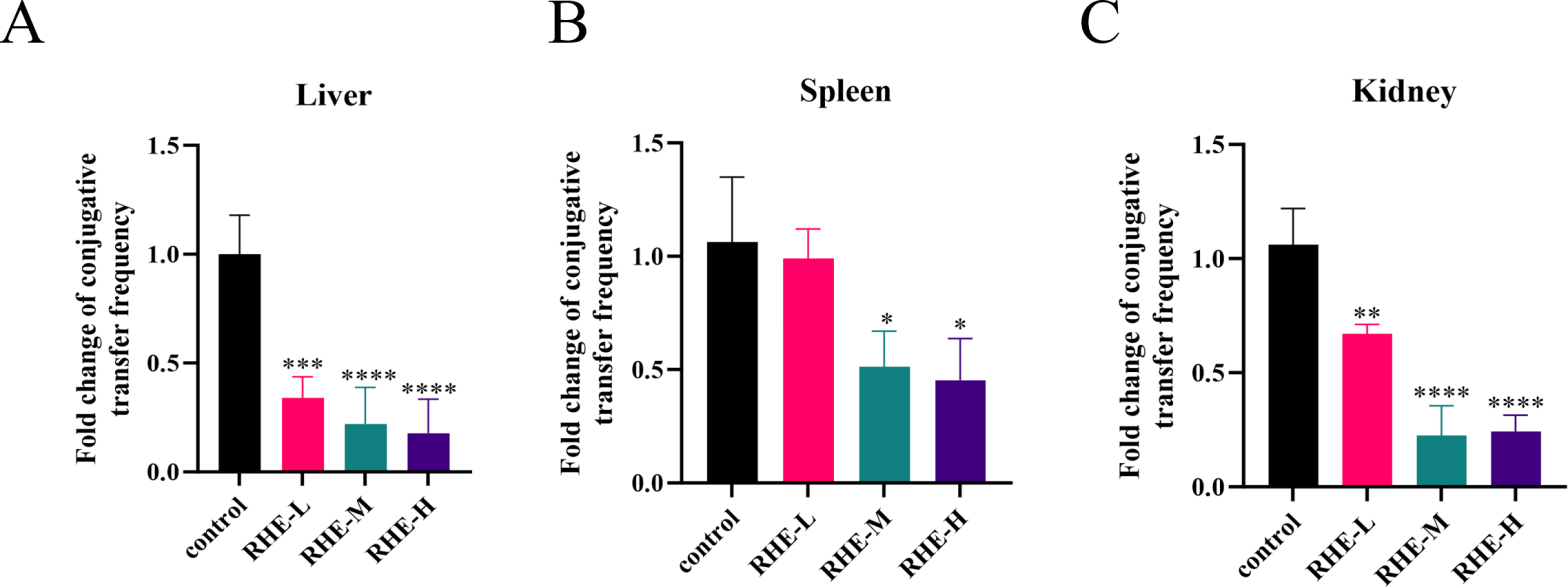
Rhein reduces the transfer frequency of the *mcr-1* gene in the liver (**A**), spleen (**B**), and kidney (**C**) *in vivo*. RHE-L, RHE 2.5 mg/kg; RHE-M, RHE 5 mg/kg; and RHE-H, RHE 10 mg/kg. Data were presented as mean ± SD (*n* = 6). Significant differences were evaluated by one-way ANOVA analysis and shown with *P < 0.05, **P < 0.01, ***P < 0.001, and ****P < 0.0001.

The murine intraperitoneal conjugation model is a well-established system for investigating *in vivo* plasmid conjugative transfer. It offers a physiologically relevant environment for observing interactions between donor and recipient bacteria, thereby more closely simulating natural conditions than *in vitro* models (28, 29). Consequently, this model was utilized to evaluate the capacity of RHE to inhibit conjugative transfer *in vivo*. Specifically, *E. coli* RCAD 0514 served as the donor strain and *E. coli* J53 as the recipient strain. Approximately 15 min following infection with a 1:1 bacterial mixture, either PBS or RHE was administered via intraperitoneal injection. After 24 h of infection, bacterial loads (donor, recipient, and transconjugants) were detected in abdominal parenchymal organs, demonstrating that these bacteria were capable of invading and infecting the liver, spleen, and kidneys. Simultaneously, the CFU counts of transconjugants and their corresponding recipients were enumerated using plates supplemented with specific antibiotics. Notably, compared with the PBS-treated group, the frequency of IncFII plasmid transfer was significantly reduced in the RHE-treated group, supporting RHE’s efficacy in inhibiting conjugative transfer in the murine infection model (Fig. 2).

### Rhein disrupts bacterial membrane integrity and depletes cellular energy

**Fig 3:**
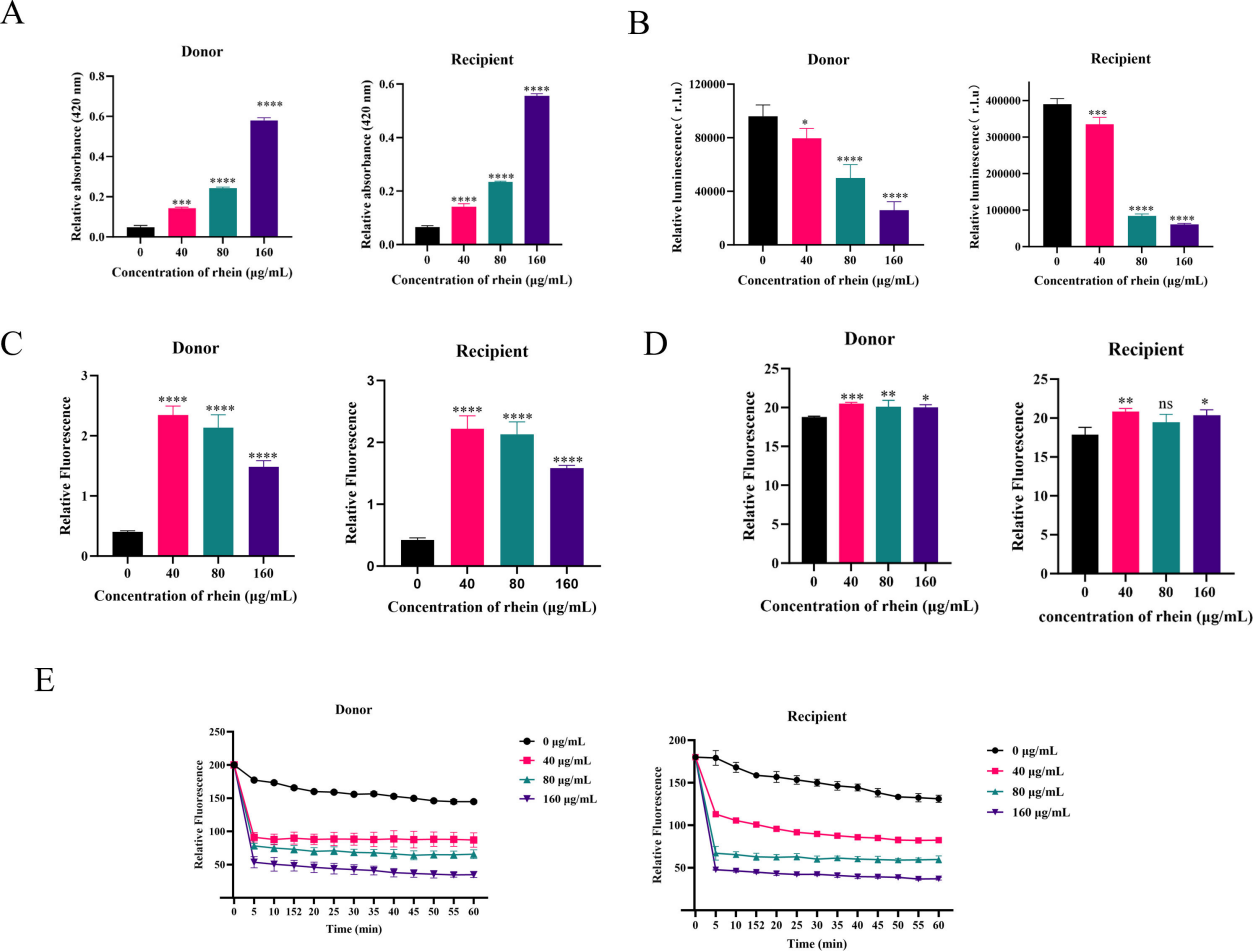
Effects of rhein on cell membrane permeability (**A**), ATP levels (**B**), ROS levels (**C**), intracellular ethidium bromide (EB) content (**D**), and proton motive force (**E**) on donor bacteria (*E. coli* DH5α containing IncFII plasmid) and recipient bacteria (*E. coli* J53). All data are presented as the mean ± SD of three independent biological replicates. Statistical significance was determined by one-way ANOVA; 40, 80, and 160 µg/mL were compared to control without rhein. *P < 0.05, **P < 0.01, ***P < 0.001, ****P < 0.0001.

Bacterial conjugation is an energy-dependent process powered by ATPases associated with the type IV secretion/conjugation machinery, and efficient transfer critically relies on intact membrane functions such as the proton motive force and membrane integrity (20, 30, 31). We therefore investigated the effect of RHE on these cellular parameters. To clarify how RHE affects the membrane properties of *E. coli*, we assessed its effects on membrane permeability in both donor and recipient strains using the ONPG assay. Results showed a concentration-dependent increase in membrane permeability in both strains, with notable effects even at 40 μg/mL (Fig. 3A). DiSC_3_(5) dye showed that RHE treatment caused obvious membrane potential dissipation, thereby disrupting PMF levels (Fig. 3E) . This finding is consistent with the inherent correlation between membrane potential and PMF homeostasis. Since ATP synthesis in *E. coli* is primarily dependent on PMF, we measured intracellular ATP levels and found that RHE exerted a dose-dependent inhibitory effect on ATP production (Fig. 3B). Cellular energy depletion has been reported to enhance susceptibility to ROS in bacteria (32). To explore this, we quantified intracellular ROS levels. RHE increased ROS production at low concentrations, while higher concentrations reduced ROS levels relative to low concentrations. Overall, however, RHE treatment elevated intracellular ROS in bacteria (Fig. 3C). Bacterial efflux pump activity relies on cellular energy. RHE-induced efflux dysfunction causes intracellular RHE accumulation, which further aggravates membrane injury and energy exhaustion and ultimately restricts plasmid conjugation. Our results indicated that RHE moderately inhibited efflux pump function (Fig. 3D). This inhibition likely promotes intracellular accumulation of RHE, further exacerbating cellular damage and potentially modulating its regulatory effects on bacterial genes, although this requires further validation. In summary, sub-inhibitory RHE treatment (40–160 μg/mL) disrupted multiple bacterial physiological processes, including increased membrane permeability, disrupted PMF and ATP production, altered ROS levels, and weakened efflux pump function. These combined damages collectively suppressed IncFII plasmid conjugation in *E. coli*.

### Rhein inhibits QS by targeting LuxS protein and impairs biofilm formation

**Fig 4:**
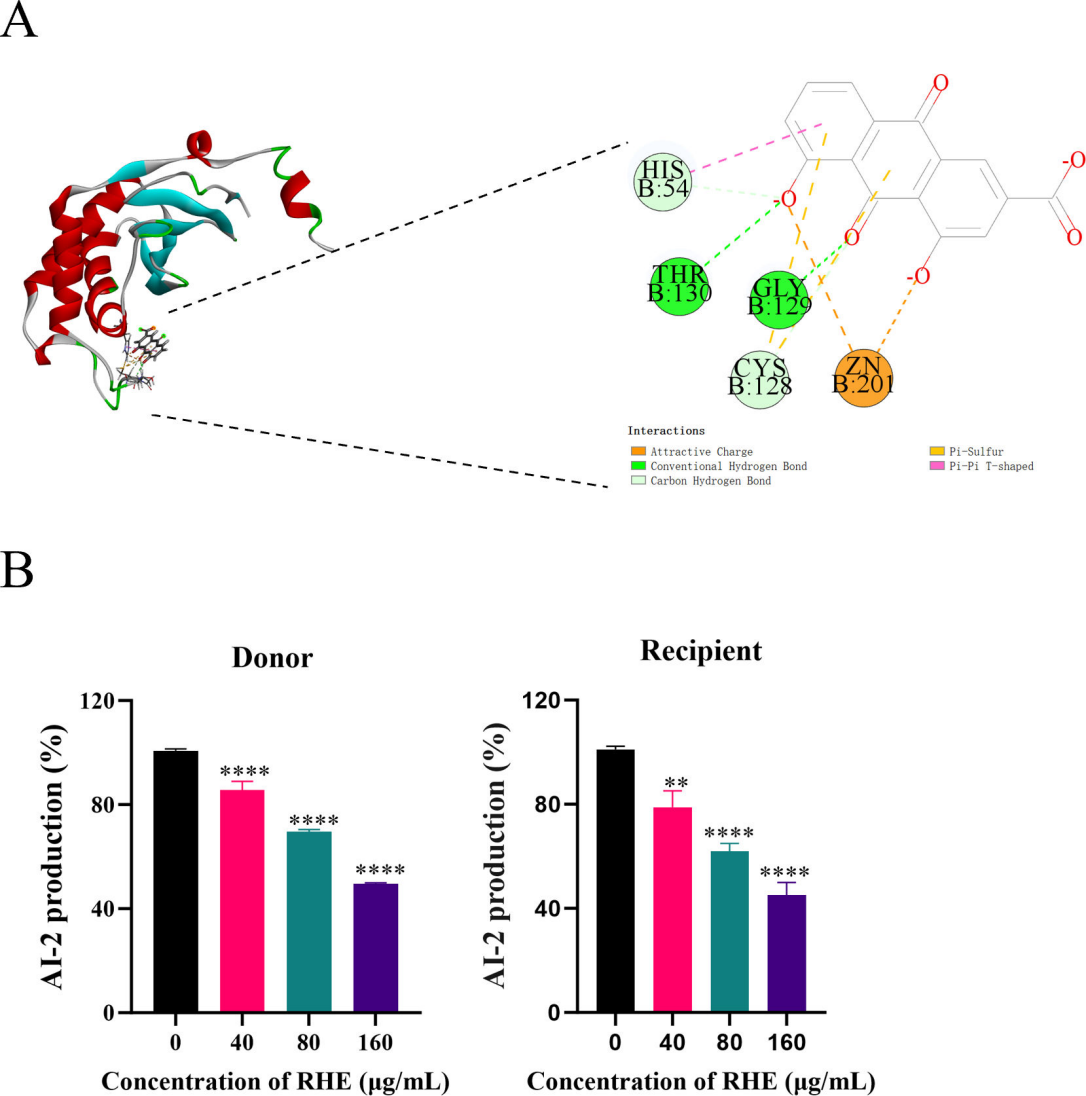
Rhein targets the LuxS/AI-2 quorum-sensing system. (**A**) Docking of RHE with LuxS protein. (**B**) RHE inhibits the production of AI-2 by donor and recipient bacteria. *P < 0.05, **P < 0.01, ***P < 0.001, ****P < 0.0001.

QS and biofilm formation are crucial for facilitating the cell-cell contact required for efficient conjugation (33). Molecular docking predicted that RHE binds to the active site of LuxS protein, a key enzyme in the synthesis of the QS signal molecule autoinducer-2 (AI-2), interacting with key residues His54, Gly129, Cys128, and Thr130 (Fig. 4A). The binding energy of the docking was −6.3 kcal/mol.

**Fig 5:**
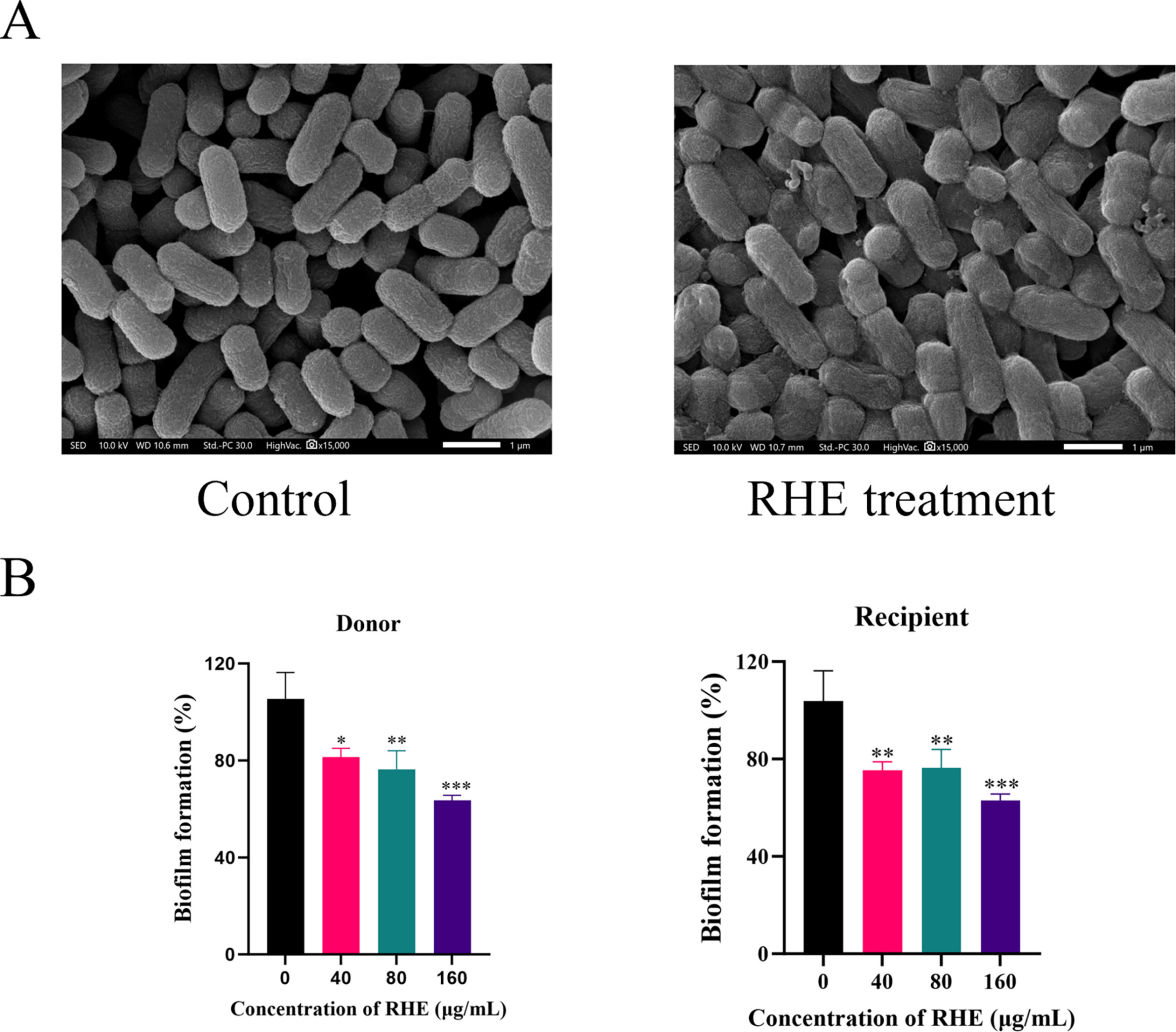
Rhein disrupts biofilm formation and cell morphology. (**A**) Scanning electron microscopy (SEM) images of bacterial cells. Scale bar = 1 µm. Magnification: ×15,000. (**B**) Quantification of biofilm formation in donor and recipient strains after treatment with 0, 40, 80, or 160 μg/mL RHE. Biofilm formation is presented as the mean ± SD of three biological replicates. Significant differences were evaluated by one-way ANOVA analysis and shown with *P < 0.05, **P < 0.01, ***P < 0.001, and ****P < 0.0001.

To validate this, we measured AI-2 production and found that RHE significantly reduced AI-2 levels in the culture supernatants of both donor and recipient strains (Fig. 4B). The inhibitory effect of RHE on conjugation was partially reversed by the addition of exogenous AI-2, confirming the role of the QS pathway (Fig. S3). As a functional consequence of QS inhibition, RHE dose dependently impaired biofilm formation by both strains (Fig. 5B). Scanning electron microscopy (SEM) revealed that RHE-treated cells exhibited morphological damage, including cell shrinkage and membrane disruption, further compromising their ability to form structured communities (Fig. 5A). Meanwhile, transcriptome sequencing results showed that RHE primarily acts on the QS system and disrupts amino acid metabolism (including serine metabolism and sulfur-containing amino acid metabolism) (15). Combined with molecular docking and the detection of AI-2 signaling molecules, it was suggested that RHE mainly targets the LuxS protein to reduce the transmission of IncFII plasmids. This mechanism may contribute to reduced conjugative efficiency in RHE-treated bacteria, likely via two pathways: reduced bacterial contact and compromised transfer machinery of bacteria treated with RHE.

### Rhein downregulates the expression of QS and biofilm-related genes

**Fig 6:**
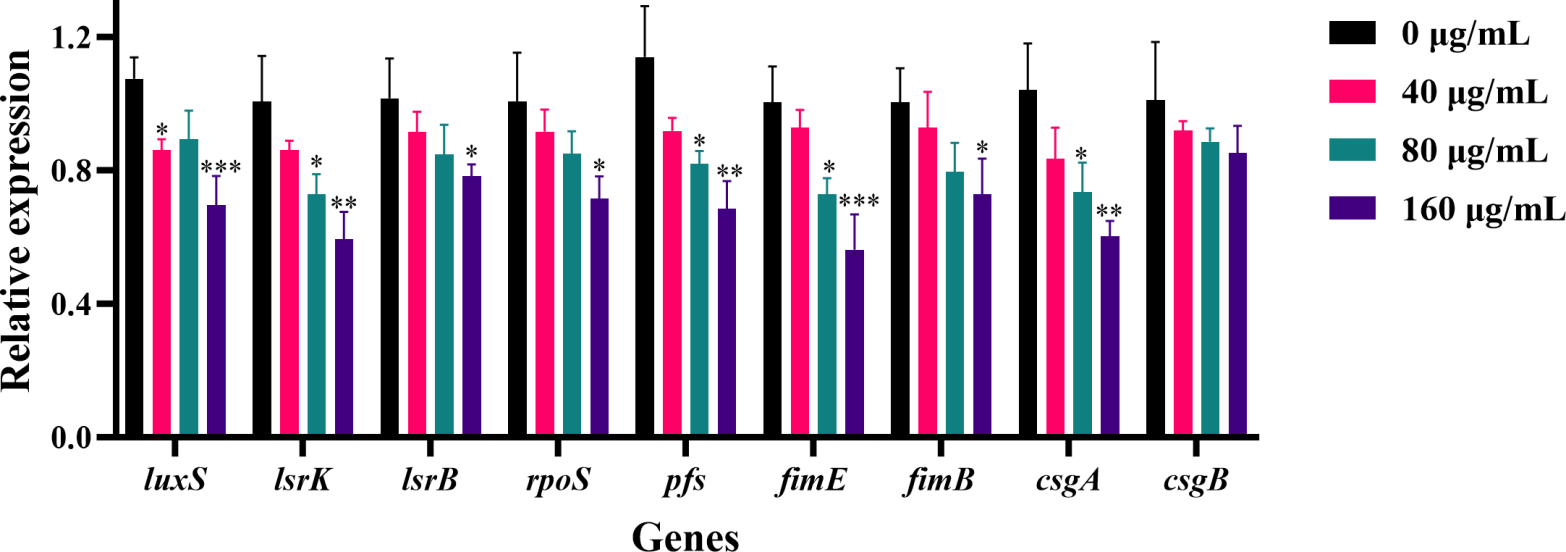
Rhein inhibits the expression of genes in the QS-biofilm regulatory network. *P < 0.05, **P < 0.01, ***P < 0.001, ****P < 0.0001.

Conjugation, a key driver of horizontal gene transfer, is regulated by the LuxS/AI-2 QS system, which coordinates adaptive behaviors, such as biofilm formation and conjugative plasmid transfer (19). Within this regulatory network, the *luxS* and *pfs* genes synergistically mediate the biosynthesis of the signaling molecule AI-2 (34). Once extracellular AI-2 accumulates to a threshold concentration, it upregulates core biofilm genes in the *csg* operon, such as *csgA* and *csgB* (35). Meanwhile, AI-2 uptake mediated by *lsrB*/*lsrK* relieves the inhibition of *lsrR*, further triggering fimbriae synthesis and F-pilus formation, which is a prerequisite for efficient conjugation (36). To further explore the mechanism of QS inhibition, we used RT-qPCR to analyze the expression of key genes in the LuxS/AI-2 regulatory network. At 160 μg/mL, RHE significantly downregulated genes spanning all layers of the network. In the signal synthesis module, *luxS* and *pfs* gene expression was reduced by 37.95% and 45.46%, respectively; in the biofilm matrix module, *csgA* gene expression decreased by 43.89%; in the signal transport module, *lsrK* and *lsrB* gene expression was inhibited by 41.20% and 23.44%, respectively; and in the phenotypic regulation module, *rpoS*, *fimE*, and *fimB* gene expression was suppressed by 29.11%, 44.22%, and 27.58%, respectively (Fig. 6). These findings demonstrate that RHE exerts multi-targeted inhibitory effects on the QS-biofilm network, encompassing signal synthesis, biofilm matrix formation, signal transduction, and phenotypic regulation. By downregulating key genes, such as *csgA*, *csgB*, and pilus assembly-related factors, RHE may disrupt the coordinated signaling cascade underlying biofilm integrity and conjugative competence. These results elucidate the mechanistic basis by which RHE impairs biofilm structure and reduces conjugation efficiency, providing molecular insights into its role in inhibiting HGT.

### Rhein modulates expression of conjugation-associated genes

**Fig 7:**
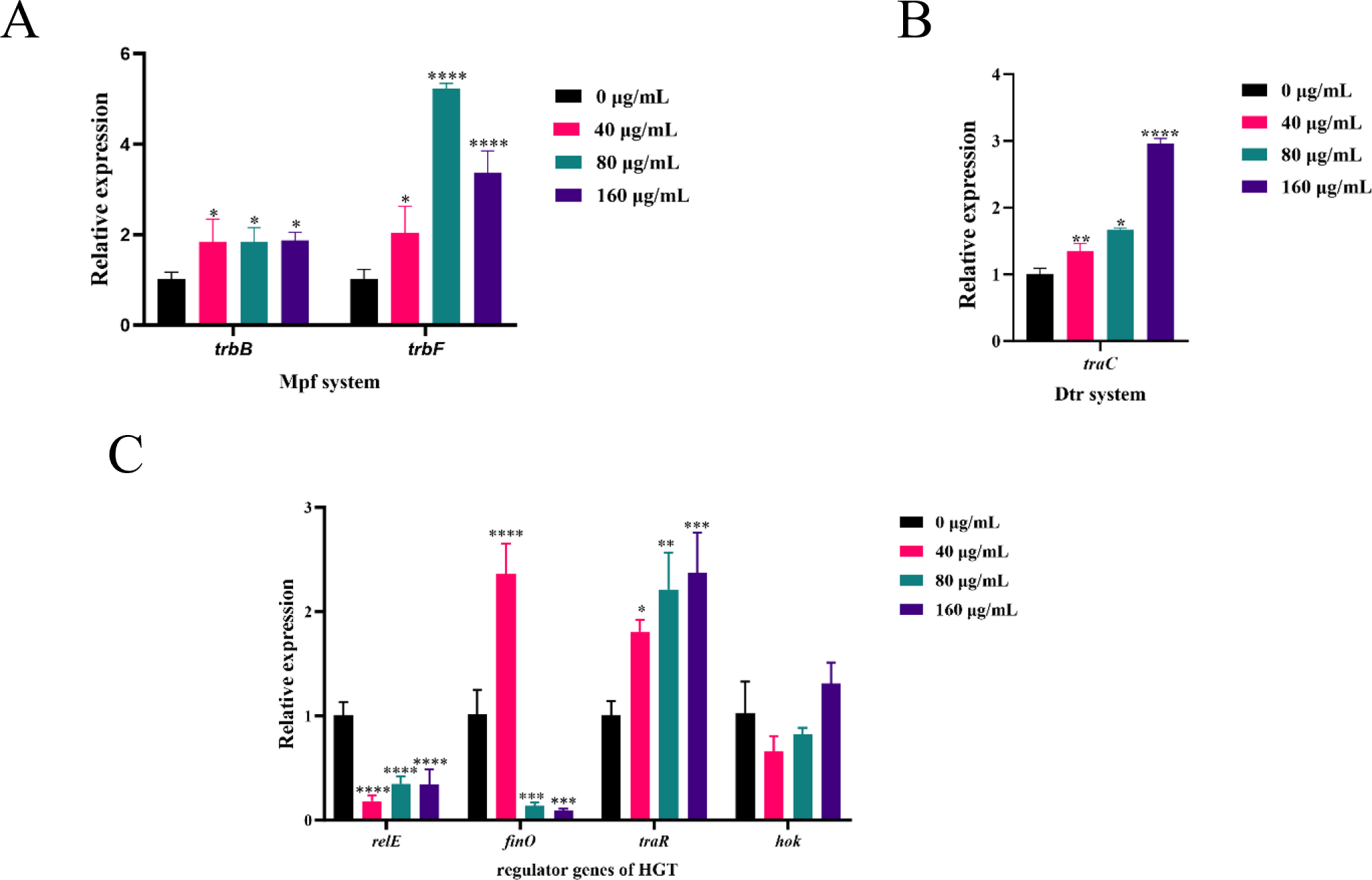
The mRNA expression of mating pair formation (Mpf) (**A**), DNA transfer and replication (Dtr) (**B**), and HGT regulatory factor (**C**)–related genes in donor bacteria (*E. coli* DH5α carrying IncFII plasmid) exposed to rhein. *P < 0.05, **P < 0.01, ***P < 0.001, ****P < 0.0001.

Given that RHE induces cellular ATP depletion, we hypothesized that energy-dependent plasmid transfer machinery could be impaired. To verify this, we assessed the expression of core conjugative transfer genes. IncFII plasmids govern their replication, mobilization, and transfer via multiple genetic modules, including DNA transfer and replication (Dtr) systems, mating pair formation (Mpf) systems, and regulatory genes (37). Among regulatory genes, *relE* and *hok* gene function as plasmid stabilization modules, while *finO* and *traR* gene constitute conjugation-specific regulatory circuits that govern the switch between low-transfer (“silent”) and high-transfer (“active”) plasmid states (38). To investigate the impact of RHE, we performed RT-qPCR analysis on key genes within the IncFII plasmid of the donor strain following RHE treatment. Targets included conjugation-repressing genes, Dtr system genes, and Mpf system genes. Notably, RHE upregulated Dtr and Mpf gene expression (Fig. 7A and B), implying that this upregulation does not counteract RHE’s conjugation-inhibitory effects. Plasmid stability is maintained by host-lethal genes (e.g., *relE* and *hok*), which suppress vertical transmission. RHE downregulated *relE* expression but had no impact on hok gene, while inhibiting *finO* gene and promoting *traR* gene expression (Fig. 7C). Functionally, Dtr genes regulate relaxosome assembly, and Mpf genes mediate trans-envelope channel formation between donor and recipient cells. Despite their essential roles in conjugation, RHE significantly induced the expression of both Dtr and Mpf genes. This counterintuitive upregulation of Dtr/Mpf genes may represent an adaptive stress response of IncFII plasmids against RHE intervention. Even though these genes are critical for conjugation, RHE blocks plasmid transfer via multiple independent pathways, including membrane damage, energy depletion, TA system disturbance, and QS inhibition.

## DISCUSSION

The horizontal transfer of antibiotic resistance genes (ARGs) via conjugative plasmids remains a major challenge in global public health, highlighting the urgent need to develop intervention strategies that inhibit ARG dissemination (39). This study demonstrates that RHE, a natural anthraquinone, reduces the conjugation of clinically relevant IncFII plasmids both *in vitro* and in a murine infection model. Our results uncovered a multi-target mechanism. RHE impairs energy-dependent conjugation machinery via membrane damage and, meanwhile, blocks QS signaling that mediates bacterial intercellular contact. This dual mechanism makes RHE superior to single-target inhibitors and a promising candidate for blocking resistance spread.

Conjugation inhibition has long relied on single-target strategies, such as blocking T4SS assembly (e.g., with azithromycin derivatives) or QS signaling (e.g., with furanones) (40–42). However, these approaches suffer from inherent drawbacks: T4SS inhibitors often exhibit narrow specificity and low inhibition efficiency, while QS inhibitors such as 4-nitropyridine-N-oxide lose efficacy under low bacterial density, which is a common scenario in clinical infections (43, 44). In contrast, RHE achieved 48–64% inhibition of IncFII plasmid transfer at 40–160 μg/mL. This efficacy persisted across varied mating times and temperatures, confirming its robustness. The anti-conjugative efficacy of RHE is primarily driven by its incapacitation of the energy-dependent T4SS machinery. RHE increases membrane permeability, collapses PMF, and reduces intracellular ATP. These damages destroy the bioenergetic basis for pilus synthesis, DNA transfer, and transconjugant generation, all of which rely on intact membranes and stable proton gradients. Crucially, this energy disruption irreversibly suppresses conjugation even when core transfer genes (*tra*, *trb*) are transcriptionally upregulated, indicating that RHE’s dominant mechanism operates post-transcriptionally via physicochemical constraints. Concomitantly, RHE modulates QS pathways by binding to LuxS, reducing AI-2, and downregulating biofilm-associated genes (*luxS*, *csgA*). This multi-target mode of action confers a distinct therapeutic advantage: simultaneous impairment of T4SS energetics and potential restriction of cell-contact opportunities enable RHE to narrow the scope for evolutionary escape. This combined targeting mode is consistent with the development strategies of novel antibacterial agents. Such strategies take physicochemical damage (e.g., membrane disruption) as the core mechanism, accompanied by the regulation of biological pathways as auxiliary antibacterial approaches. This dual-pronged approach is inherently less susceptible to the development of drug resistance than single-target inhibitors.

Notably, the interplay between QS and membrane/energy disruption amplifies inhibition. RHE disrupts QS-regulated biofilms by suppressing the *csgA gene* and AI-2 expression, thereby reducing cell-cell contact. Concurrently, membrane damage and ATP loss directly block T4SS-mediated DNA transfer. The observed two-fold action recapitulates the enhanced efficacy seen when combining quorum quenching agents with membrane-targeting compounds, confirming that RHE possesses a distinct mechanistic advantage for inhibiting conjugation within biofilms. A striking observation was the upregulation of Dtr system genes and Mpf system genes despite reduced conjugation efficiency. Such transcriptional upregulation represents a typical plasmid stress response. With complete adaptive systems, IncFII plasmids can sense energy deficiency and compensate by enhancing the expression of transfer-related genes (45).

Several conjugation inhibitors have been reported, including unsaturated fatty acids (2-HDA) (46), nucleoside analogs (AZT) (47), synthetic small molecules, and natural products such as baicalin and dihydroartemisinin (33, 48). Most of these agents exert their effects through a single mechanism, for instance, targeting the TrwD ATPase, interfering with QS, or disrupting the type IV secretion system (33, 46, 48). In comparison, RHE exerts a unique dual-mode action. It damages bacterial membrane structure, reduces energy metabolism, and blocks the LuxS/AI-2 QS pathway, thereby effectively inhibiting IncFII plasmid conjugation. Furthermore, RHE significantly reduces *mcr-1* transmission both *in vitro* and *in vivo*, whereas most documented inhibitors lack confirmed *in vivo* activity. With its multi-target mode of action, low tendency to induce resistance, and natural source, RHE exhibits distinct advantages over most currently available conjugation inhibitors and serves as a promising candidate for curbing the dissemination of antibiotic resistance.

Existing MCR-1 inhibitors, such as polymyxin B derivatives, primarily act to restore colistin sensitivity against *mcr-1*-positive pathogens but fail to suppress the horizontal gene transfer (HGT) of the *mcr-1* gene (49). By contrast, RHE exerts a dual function: besides restoring the susceptibility of resistant strains to colistin, it also reduces the transmission frequency of the *mcr-1* gene by approximately 79%–86%. This unique dual action, which simultaneously inhibits *mcr*-1 gene dissemination and enhances colistin antibacterial activity, confers two pivotal clinical benefits: curbing the spread of colistin resistance and improving antibiotic therapeutic efficacy. Notably, this dual advantage distinguishes RHE from most anti-resistance (AR) interventions, which usually target either the resistance mechanism itself or HGT, but rarely both (15).

### Conclusion

In conclusion, this study provides experimental evidence that rhein (RHE) effectively reduces the conjugative transfer of IncFII plasmids in *E. coli* under both *in vitro* and *in vivo* conditions. Mechanistically, RHE blocks HGT through three sequential pathways: impaired membrane integrity and PMF, decreased intracellular ATP production, and suppressed QS activity and biofilm formation. Collectively, these multi-modal effects act synergistically to limit plasmid dissemination. These findings underscore the translational potential of RHE as a promising therapeutic agent to combat the global spread of antibiotic resistance.

## Supplementary Material

Reviewer comments

## Data Availability

All data generated or analyzed during this study are included in this published article (and its Supplemental material).

## SUPPLEMENTAL MATERIAL

Supplemental material spectrum.03399-25-s0001.docx Tables S1 to S7; Fig. S1 to S3.

OPEN PEER REVIEW reviewer-comments.pdf An accounting of the reviewer comments and feedback.

ASM does not own the copyrights to Supplemental Material that may be linked to, or accessed through, an article. The authors have granted ASM a non-exclusive, world-wide license to publish the Supplemental Material files. Please contact the corresponding author directly for reuse.
