# Supplementary material for: Rhein reduces conjugation of IncFII-type plasmids in Escherichia coli and mitigates the spread of antibiotic resistance genes
Source: Microbiol Spectr. 2026 Jul 6;14(8):e03399-25. doi: 10.1128/spectrum.03399-25 (PMC13435978; doi:10.1128/spectrum.03399-25)
Supplement: Supplementary file 1 [file reviewer-comments.pdf]

AMERICAN SOCIETY FOR MICROBIOLOGY | Microbiology Spectrum

Rhein Reduces Conjugation of IncFII-Type Plasmids in *Escherichia coli* and Mitigates the Spread of Antibiotic Resistance Genes

Nannan wang, Haitao Ma, Hanyan Cheng, Xu Yang, Long Liu, Dekang Zhu, Shaqiu Zhang, Xun Zhou, Renyong Jia, Yuanfeng Zou, Lixia Li, Xu Song, and yin zhongqiong

Corresponding Author(s): yin zhongqiong, Sichuan Agricultural University

Review Timeline:

Submission Date:	October 20, 2025
Editorial Decision:	December 31, 2025
Revision Received:	February 26, 2026
Editorial Decision:	April 22, 2026
Revision Received:	May 22, 2026
Accepted:	June 13, 2026

Editor: Jian Peng

Reviewer(s): The reviewers have opted to remain anonymous.

Transaction Report:

(Note: With the exception of the correction of typographical or spelling errors that could be a source of ambiguity, letters and reports are not edited. The original formatting of letters and referee reports may not be reflected in this compilation.)

DOI: https://doi.org/10.1128/spectrum.03399-25

1st Editorial Decision December 31, 2025]

Re: Spectrum03399-25 (**Rhein Inhibits Conjugation of IncFII-Type Plasmids in *Escherichia coli* and Mitigates the Spread of Antibiotic Resistance Genes**)

Dear Prof. Zhongqiong Yin:

Thank you for the privilege of reviewing your work. Below you will find my comments, instructions from the Spectrum editorial office, and the reviewer comments.

Please return the manuscript within 60 days; if you cannot complete the modification within this time period, please contact me. If you do not wish to modify the manuscript and prefer to submit it to another journal, notify me immediately so that the manuscript may be formally withdrawn from consideration by Spectrum.

Revision Guidelines
To submit your modified manuscript, log into the submission site at https://spectrum.msubmit.net/cgi-bin/main.plex. Go to Author Tasks and click the appropriate manuscript title to begin. The information you entered when you first submitted the paper will be displayed; update this as necessary. Note the following requirements:

• Upload point-by-point responses to the issues raised by the reviewers in a file named "Response to Reviewers," NOT in your cover letter.
• Upload a compare copy of the manuscript (without figures) as a "Marked-Up Manuscript" file.
• Upload a clean .DOC/.DOCX version of the revised manuscript and remove the previous version.
• Each figure must be uploaded as a separate, editable, high-resolution file (TIFF or EPS preferred), and any multipanel figures must be assembled into one file.
• Any supplemental material intended for posting by ASM should be uploaded with their legends separate from the main manuscript. You can combine all supplemental material into one file (preferred) or split it into a maximum of 10 files with all associated legends included.

For complete guidelines on revision requirements, see our Submission and Review Process webpage. Submission of a paper that does not conform to guidelines may delay acceptance of your manuscript.

Data availability: ASM policy requires that data be available to the public upon online posting of the article, so please verify all links to sequence records, if present, and make sure that each number retrieves the full record of the data. If a new accession number is not linked or a link is broken, provide Spectrum production staff with the correct URL for the record. If the accession numbers for new data are not publicly accessible before the expected online posting of the article, publication may be delayed; please contact production staff (Spectrum@asmusa.org) immediately with the expected release date.

Publication Fees: For information on publication fees and which article types are subject to charges, visit our website. If your manuscript is accepted for publication and any fees apply, you will be contacted separately about payment during the production process; please follow the instructions in that e-mail. Arrangements for payment must be made before your article is published.

ASM Membership: Corresponding authors may join or renew ASM membership to obtain discounts on publication fees. Need to upgrade your membership level? Please contact Customer Service at Service@asmusa.org.

The ASM Journals program strives for constant improvement in our submission and publication process. Please tell us how we can improve your experience by taking this quick Author Survey.

Thank you for submitting your paper to Spectrum.

Sincerely,
Jian Peng
Editor
Microbiology Spectrum

Reviewer #1 (Comments for the Author):

This study confirms that rhein (RHE) effectively inhibits conjugative transfer of E. coli IncFII-type plasmids both in vitro and in mouse infection models. It reveals a synergistic mechanism whereby RHE disrupts bacterial membrane integrity, depletes energy reserves, and interferes with the quorum sensing system, thereby slowing the spread of antibiotic resistance genes-particularly mcr-1. This provides a novel and viable strategy for addressing the antibiotic resistance crisis.

This manuscript describes a very nice study that will be of general interest to the readers of MS. However, the following revisions in manuscript should be revised before it is accepted.
Comments:
The description of Rhein should be more detailed.

Line 67-68 With the approval of drugs such as cefuroxime, daptomycin, and canithiazole for clinical use, it is no longer appropriate to refer to colistin as a "last-line antibiotic.

Line 94-95 No relevant references provided.

Line 109-110 Dose E. coli BH126 carry the IncFII plasmid? Is the mcr-1 gene in both strains located on IncFII plasmid?

Line 127-128 Please add the detailed RHE concentration gradient here.

Line 156 E. coli RCAD 0514 is used as the donor in vivo assay, yet in the in vitro conjugation experiment, the DH5α/IncFII plasmid serves as the donor. Why can't the same donor be used?

Line 486-491 This description is a repetition of the content in the Introduction section and is redundant here.

Reviewer #2 (Comments for the Author):

In this paper, Wang et al. investigated the effects of rhein, a natural product compound found in rhubarb species, on plasmid transmission in E. coli, and its mechanism(s) of action. The spread of AMR genes through conjugation in bacterial pathogens is a key contributing factor to the increasing rates of treatment failure. They report that sub-inhibitory concentrations of rhein reduced in vitro conjugation of IncFII and mcr-1 carrying plasmids in E. coli. They also report that rhein reduces the in vivo transmission of IncFII plasmid carrying mcr-1 between E. coli in a mice model. To determine the mechanism by which rhein reduced plasmid conjugation, they investigated its effect on bacterial physiology and processes, including membrane permeability and potential, ATP quantification, ROS generation, efflux pump activity, biofilm formation, and plasmid and QS gene expression.

My main concerns are that some of the findings are overstated and therefore need to be softened. For example, the authors state in the title that rhein inhibits plasmid transfer, but their data is represented as fold-changes in conjugative frequency, which often does not translate to significant changes in conjugation frequency in a given bacterial population. Conjugation frequency data is often expressed in log-scale, where a reduction of at least >10-fold (log-fold) would be considered a significant reduction. At the very least, the authors need to provide raw conjugation frequency data in the supplementary and soften the wording 'inhibits' throughout the paper.

Regarding the in vivo mice data, I have some comments:
• I'm quite surprised that E. coli J53, a K-12 derivative, was able to invade and infect the liver, spleen, and kidneys. Were the mice germ-free or cleared of their microbiota by antibiotic treatment? If so, this should be stated somewhere.
• The dose of donor and recipient E. coli strains were very high, so how clinically relevant would that be?
• Is rhein cytotoxic? Was this tested beforehand or has it been determined previously?
• Also, there are no ethics statement on how the mice experiments were carried out. This needs to be included.

Em dashes (-) are used throughout the paper, which to me strongly suggests the use of AI. If this is the case, then this should be appropriately mentioned and acknowledged.

In Figure 3D, what is this data showing? Is this the final fluorescence value of ethidium bromide after 60 min? If so, a lower relative fluorescence would indicate less ethidium bromide accumulation within cells, suggesting efflux pump potentiation rather than inhibition. This would suggest that rhein does not inhibit/reduce efflux pump activity.

Figure legends need more detail; the readers should be able to understand the figure without referring to the text. For example, in figure 2, what is the L, M, and H dose? This sort of detail is needed in the figure legend.

Does rhein affect fluorescence of any of the dyes used in the study? Just looking through the literature, rhein seems to have a maximum excitation wavelength of 440 nm and an emission maximum of 520 nm. This would likely interfere with the fluorescence of some dyes used in this study.

The docking results are very superficial. For example, there are no predicted binding energy data or images of the predicted docking sites.

The authors need to double-check all references to make sure that it matches what they are claiming to cite.

Specific comments:
Line 22: Why is rhein a first-in-class inhibitor? There are many other plasmid conjugation inhibitors reported in the literature.
Line 37: The 'Importance' section reads like the abstract but just shortened. I think it needs to be re-written to indicate the significance of their research to a broad audience.
Line 52: The use of 'ecosystem stability' is quite vague here. What do you mean?
Line 57: This study (https://journals.asm.org/doi/full/10.1128/msphere.00170-23) should referenced here as it shows that growth in a biofilm promotes plasmid conjugation.
Line 69: CTX-M-55 does not need to be italicised.
Line 92: What does 'anti-resistance agent' mean? I think it needs to be clarified.
Line 95: Is quorum-sensing necessary for plasmid conjugation? Or does it just increase/promote transfer frequency?
Line 114: What is rhein dissolved in?
Line 134: Overnight does not need to be capitalised.
Line 149: What does 'in triplicate' mean? Were these biological replicates? Was this on the same day or on different days?
Line 150: Should be 'In vivo conjugation assay'
Lien 160: What dose of avertin was used?
Line 167: Why was ONPG hydrolysis used to measure membrane permeability? I'm familiar with PI or NPN uptake being used to determine this in most studies.
Line 178-179: This should be changed to 'Ethidium bromide accumulation assay', as you are not directly measuring efflux, but rather accumulation of ethidium bromide. Also, in the Song et al. paper, I could not find the ethidium bromide accumulation assay method. Please double-check this reference and cite a more appropriate reference.
Line 202: Reference the Livak method here.
Line 314: I could not find anything about bacterial conjugation in this citation (number 26).
Line 325: This sentence needs a reference
Line 361: Change 'confirmed' to 'suggested'
Line 382: csgA and csgB do not need to be capitalised.
Figure 7: Gene names should be italicised.
Line 442: Change 'potently inhibits' to 'reduces'
Line 445: Change 'cripples' to 'disrupts'
Line 485: This claim needs a reference to support it.

Sir,

This study confirms that rhein (RHE) effectively inhibits conjugative transfer of *E. coli* IncFII-type plasmids both in vitro and in mouse infection models. It reveals a synergistic mechanism whereby RHE disrupts bacterial membrane integrity, depletes energy reserves, and interferes with the quorum sensing system, thereby slowing the spread of antibiotic resistance genes—particularly *mcr-1*. This provides a novel and viable strategy for addressing the antibiotic resistance crisis.

This manuscript describes a very nice study that will be of general interest to the readers of MS. However, the following revisions in manuscript should be revised before it is accepted.

Comments:

The description of Rhein should be more detailed.

Line 67-68 With the approval of drugs such as cefuroxime, daptomycin, and canithiazole for clinical use, it is no longer appropriate to refer to colistin as a "last-line antibiotic.

Line 94-95 No relevant references provided.

Line 109-110 Dose *E. coli* BH126 carry the IncFII plasmid? Is the *mcr-1* gene in both strains located on IncFII plasmid?

Line 127-128 Please add the detailed RHE concentration gradient here.

Line 156 *E. coli* RCAD 0514 is used as the donor in vivo assay, yet in the in vitro conjugation experiment, the DH5α/IncFII plasmid serves as the donor. Why can't the same donor be used?

Line 486-491 This description is a repetition of the content in the Introduction section and is redundant here.

Dear Editor and Reviewers,

We appreciate the opportunity to revise our manuscript titled " **Rhein Reduces Conjugation of IncFII-Type Plasmids in *Escherichia coli* and Mitigates the Spread of Antibiotic Resistance Genes**" **(Manuscript ID:Spectrum03399-25)** and are grateful for the insightful comments provided by the reviewers. Those comments are all valuable and very helpful for revising and improving our paper, as well as the important guiding significance to our researches. In the following, we have provided detailed responses to each of the reviewers' comments. Revised portion is highlighted in yellow the paper. Additionally, we have conducted a comprehensive revision of the entire manuscript. In this response letter, the reviewers' comments are presented in black, and our corresponding changes and additions to the manuscript are highlighted in blue text. We have tried our best to make all the revisions clear, and we hope that the revised manuscript meets the requirements for publication.

Reviewer #1 (Comments for the Author):

This study confirms that rhein (RHE) effectively inhibits conjugative transfer of E. coli IncFII-type plasmids both in vitro and in mouse infection models. It reveals a synergistic mechanism whereby RHE disrupts bacterial membrane integrity, depletes energy reserves, and interferes with the quorum sensing system, thereby slowing the spread of antibiotic resistance genes-particularly mcr-1. This provides a novel and viable strategy for addressing the antibiotic resistance crisis.

This manuscript describes a very nice study that will be of general interest to the readers of MS. However, the following revisions in manuscript should be revised before it is accepted.

Comments:

The description of Rhein should be more detailed.

Response: Thanks for your valuable comments. We have revised the description of rhein to be more detailed “Rhein (4,5’-dihydroxyanthraquinone-2-carboxylic acid, RHE), a naturally occurring anthraquinone derivative isolated from medicinal plants

including *Rheum palmatum*, *Cassia obtusifolia*, *Polygonum multiflorum* and *Aloe vera*, has emerged as a promising candidate for pharmacological intervention in recent years(12). Extensive research has corroborated its potent hepatoprotective, neuroprotective, anticancer and anti-inflammatory bioactivities(13). Beyond these well-characterized properties, a growing body of evidence indicates that RHE exerts remarkable antimicrobial efficacy against a broad spectrum of Gram-positive and Gram-negative bacteria, such as *Bacillus megaterium*, *Staphylococcus aureus* and *Cutibacterium acnes*(12). ” Line 90-100

12. Folliero V, Annunziata FD, Roscetto E, et al. Rhein : A novel antibacterial compound against Streptococcus mutans infection. MICROBIOL RES. 2022; 261.

13. Wang H, Yang D, Li L, et al. Anti-inflammatory Effects and Mechanisms of Rhein, an Anthraquinone Compound, and Its Applications in Treating Arthritis: A Review. NAT PRODUCT BIOPROSP. 2020; 10: 445-52.

Line 67-68 With the approval of drugs such as cefuroxime, daptomycin, and canithiazole for clinical use, it is no longer appropriate to refer to colistin as a "last-line antibiotic.

Response: Thanks for your valuable comments. We have revised the expression related to colistin, which is now stated as “Colistin, an important antibiotic for treating carbapenem-resistant infections, is becoming increasingly ineffective due to the spread of the *mcr* genes.” Line 65-66

Line 94-95 No relevant references provided.

Response: Thanks for your valuable comments. We have added relevant references：

15. Wang N, Yang D, Cao L, et al. Rhein restores the sensitivity of *mcr-1* carrying multidrug-resistant *Escherichia coli* to colistin. FRONT MICROBIOL. 2025; 16: 1586553.

16. Zhang Y, Ma Q, Su B, et al. A study on the role that quorum sensing play in

antibiotic-resistant plasmid conjugative transfer in *Escherichia coli*. ECOTOXICOLOGY. 2018; 27: 209-16.

17. Xiao X, Zeng F, Li R, et al. Subinhibitory concentration of colistin promotes the conjugation frequencies of *mcr-1* - and *bla*$_{NDM-5}$ -positive plasmids. MICROBIOL SPECTR. 2022; 10.

Line 109-110 Dose E. coli BH126 carry the IncFII plasmid? Is the mcr-1 gene in both strains located on IncFII plasmid?

Response: We sincerely thank the reviewers for their valuable feedback. *E. coli* BH126 also carries an IncFII plasmid. In these two strains, the *mcr-1* gene is not located on the IncFII plasmid, but on a non-conjugative plasmid.

Line 127-128 Please add the detailed RHE concentration gradient here.

Response: Thanks for your valuable comments. It has been added. "The bacterial suspensions were incubated at 37°C with shaking (200 rpm) in the presence of RHE (0, 40, 80 and 160 μg/mL)" Line 136

Line 156 E. coli RCAD 0514 is used as the donor in vivo assay, yet in the in vitro conjugation experiment, the DH5α/IncFII plasmid serves as the donor. Why can't the same donor be used?

Response: Thanks for your valuable comments. *E. coli* DH5α harboring the IncFII plasmid exhibited weaker in vivo colonization ability than the wild-type *E. coli* strain RCAD 0514, with a relatively low in vivo conjugation efficiency. Accordingly, *E. coli* RCAD 0514 was employed as the donor strain. The *mcr-1* gene in *E. coli* RCAD 0514 was carried by its IncFII plasmid, which mediated the conjugative transfer of the strain. For this reason, *E. coli* RCAD 0514 was designated as the donor bacterium for in vivo conjugation assays. Furthermore, the transfer of the *mcr-1* gene in *E. coli* RCAD 0514 was dependent on the conjugative capacity of the IncFII plasmid. This strain could also be used to evaluate the effects of rhein on the transfer capability of

the IncFII vector and the dissemination of the *mcr-1* gene.

Line 486-491 This description is a repetition of the content in the Introduction section and is redundant here.

Response: Thanks for your valuable comments. We have rewritten the paragraph "Existing MCR-1 inhibitors, such as polymyxin B derivatives, primarily act to restore colistin sensitivity against *mcr-1*-positive pathogens but fail to suppress the horizontal gene transfer (HGT) of the mcr-1 gene(46). By contrast, RHE exerts a dual function: besides restoring the susceptibility of resistant strains to colistin, it also reduces the transmission frequency of the *mcr-1* gene by approximately 79%-86%. This unique dual action, which simultaneously inhibits *mcr*-1 gene dissemination and enhances colistin antibacterial activity, confers two pivotal clinical benefits: curbing the spread of colistin resistance and improving antibiotic therapeutic efficacy. Notably, this dual advantage distinguishes RHE from most anti-resistance (AR) interventions, which usually target either the resistance mechanism itself or HGT, but rarely both(15)" Line 509-518

Reviewer #2 (Comments for the Author):

In this paper, Wang et al. investigated the effects of rhein, a natural product compound found in rhubarb species, on plasmid transmission in E. coli, and its mechanism(s) of action. The spread of AMR genes through conjugation in bacterial pathogens is a key contributing factor to the increasing rates of treatment failure. They report that sub-inhibitory concentrations of rhein reduced in vitro conjugation of IncFII and mcr-1 carrying plasmids in E. coli. They also report that rhein reduces the in vivo transmission of IncFII plasmid carrying mcr-1 between E. coli in a mice model. To determine the mechanism by which rhein reduced plasmid conjugation, they investigated its effect on bacterial physiology and processes, including membrane permeability and potential, ATP quantification, ROS generation, efflux pump activity,

biofilm formation, and plasmid and QS gene expression.

My main concerns are that some of the findings are overstated and therefore need to be softened. For example, the authors state in the title that rhein inhibits plasmid transfer, but their data is represented as fold-changes in conjugative frequency, which often does not translate to significant changes in conjugation frequency in a given bacterial population. Conjugation frequency data is often expressed in log-scale, where a reduction of at least >10-fold (log-fold) would be considered a significant reduction. At the very least, the authors need to provide raw conjugation frequency data in the supplementary and soften the wording 'inhibits' throughout the paper.

Response: Thank you for your valuable feedback. We have provided the original conjugation frequency data for supplementary materials (Table S4-S7) and softened the language expression throughout the full text.

Regarding the in vivo mice data, I have some comments:

I'm quite surprised that E. coli J53, a K-12 derivative, was able to invade and infect the liver, spleen, and kidneys. Were the mice germ-free or cleared of their microbiota by antibiotic treatment? If so, this should be stated somewhere.

Response: Thank you for your valuable feedback. A high-concentration suspension of the target strain was administered via intraperitoneal injection, with the strain demonstrating short-term survival in the murine liver, kidneys, and spleen. Notably, the specific pathogen-free (SPF) grade mice used in this study were procured from Chengdu Dashuo Experimental Animal Co., Ltd., and this information has been incorporated into the manuscript. Line 130-131；Line167

The dose of donor and recipient E. coli strains were very high, so how clinically relevant would that be?

Response: We sincerely thank the reviewers for their valuable feedback. In the peritonitis experiment of studying the conjugation transfer of drug-resistant plasmids, a mixed bacterial solution (donor bacteria+recipient bacteria) of 1×10^8 - 5×10^8 CFU is commonly injected to ensure sufficient contact probability between the donor and

recipient bacteria in the abdominal microenvironment, thereby detecting quantifiable conjugates. Simulating the local high bacterial infection microenvironment of severe clinical peritonitis, this dose is consistent with the bacterial concentration range of the abdominal infection site and can meet the detection needs of drug-resistant plasmid conjugation transfer; Meanwhile, this model can be used to evaluate the inhibitory effect of intervention strategies on the spread of drug-resistant genes under extreme infection conditions, providing experimental evidence for the prevention and control of severe clinical infections.

• Is rhein cytotoxic? Was this tested beforehand or has it been determined previously?

Response: Thank you for your valuable feedback. Our previous research has confirmed that 10 mg/kg rhein is harmless to mice, and the drug concentration used in this study had no significant toxicity to mice[1]. In the determination of in vitro cytotoxicity, rhein showed no toxicity to cells at a concentration of 40 μg/mL.

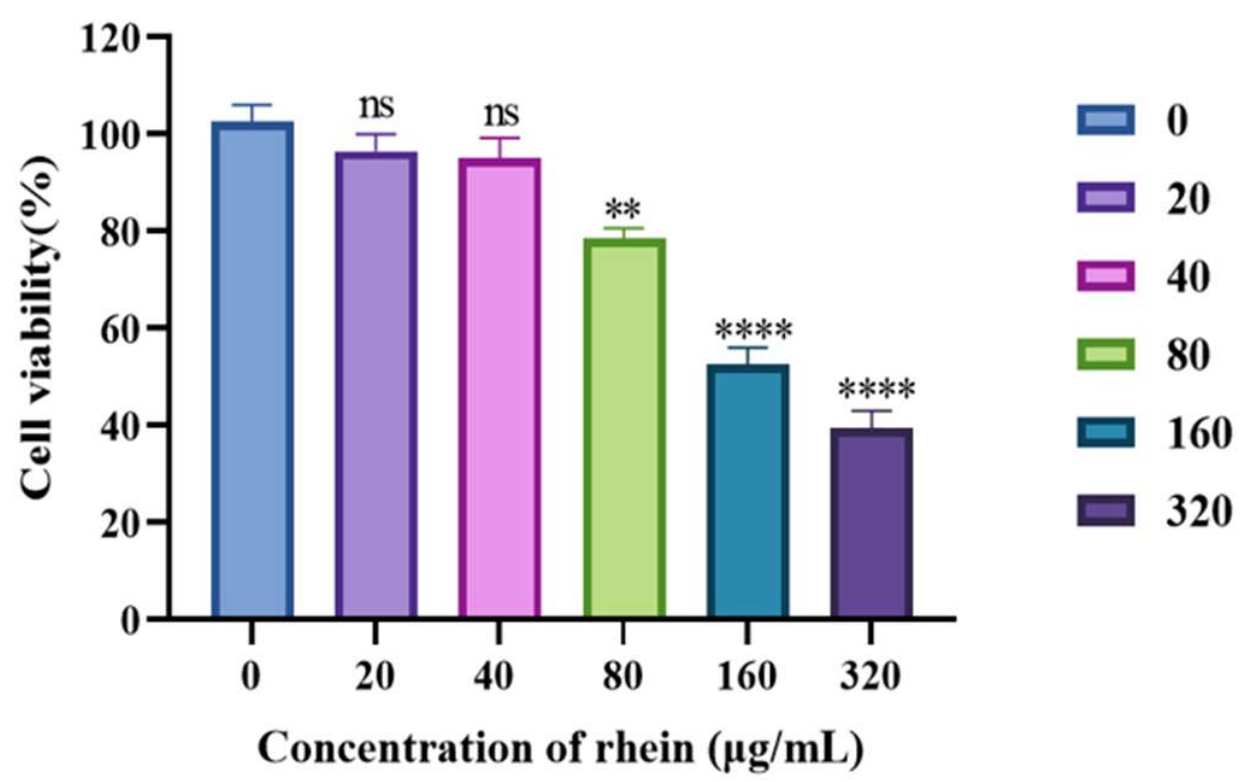

[1] Wang N, Yang D, Cao L, et al. Rhein restores the sensitivity of mcr-1 carrying multidrug-resistant Escherichia coli to colistin. FRONT MICROBIOL. 2025; 16: 1586553.

• Also, there are no ethics statement on how the mice experiments were carried out. This needs to be included.

Response: We sincerely thank the reviewers for their valuable feedback. It has been added. “All mice were housed in the Animal Center of the College of Veterinary

Medicine, Sichuan Agricultural University under standard conditions with free access to food and water. The light was from 8:00 am to 8:00 pm, with the temperature kept at 22±1℃ and humidity at 40-70%. All experimental procedures involving animals were in accordance with the guidelines of the Animal Care and Use Committee of Sichuan Agricultural University. All animal experiments were performed independently of each other with different cohorts of mice." Line 159-165

Em dashes (-) are used throughout the paper, which to me strongly suggests the use of AI. If this is the case, then this should be appropriately mentioned and acknowledged.

Response: Thank you for your valuable feedback. It has been added. "The authors acknowledge the use of AI o assist in language polishing of articles." Line 530

In Figure 3D, what is this data showing? Is this the final fluorescence value of ethidium bromide after 60 min? If so, a lower relative fluorescence would indicate less ethidium bromide accumulation within cells, suggesting efflux pump potentiation rather than inhibition. This would suggest that rhein does not inhibit/reduce efflux pump activity.

Response: Thanks for your valuable comments. Upon careful examination, we sincerely apologize for the incorrect description of the method for determining intracellular ethidium bromide (EB) content in the Materials and Methods section. We apologize for any inconvenience caused and have now corrected this error. In addition, the plotting data of Figure 3D were confused with other datasets, which resulted in inconsistencies between the presented results and the corresponding experimental data. Figure 3D has been revised accordingly. Line 192-196 and Figure 3D

D

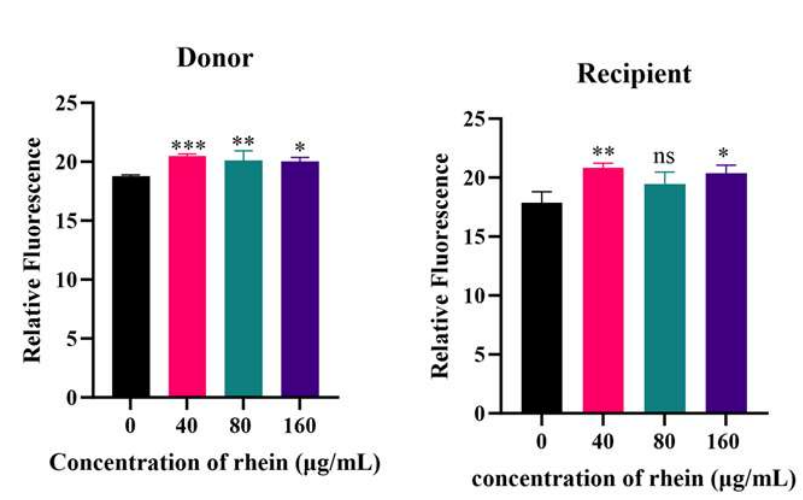

Figure legends need more detail; the readers should be able to understand the figure without referring to the text. For example, in figure 2, what is the L, M, and H dose? This sort of detail is needed in the figure legend.

Response: Thanks for your valuable comments. It has been modified.

"**Figure 2** Rhein reduces the transfer frequency of the mcr-1 gene in the liver (A), spleen (B), and kidney (C) in vivo. RHE-L,RHE 2.5 mg/kg; RHE-M,RHE 5 mg/kg; RHE-H, RHE 10 mg/kg. Data were presented as mean ± SD (n = 6). Significant differences were evaluated by one-way ANOVA analysis and shown with *P<0.05, **P< 0.01, ***P< 0.001, ****P< 0.0001. Line 322-323

Figure 3 Effects of rhein on cell membrane permeability(A), ATP levels(B), ROS levels(C), intracellular ethidium bromide (EB) content (D) and Proton motive forcin(E) on donor bacteria (*E. coli* DH5α containing IncFII plasmid) and recipient bacteria (*E. coli* J53). Data were presented as mean ± SD from three biological replicates. Significant differences were evaluated by one-way ANOVA analysis and shown with *P<0.05, **P< 0.01, ***P< 0.001, ****P< 0.0001. Line 358-362 "

Does rhein affect fluorescence of any of the dyes used in the study? Just looking through the literature, rhein seems to have a maximum excitation wavelength of 440 nm and an emission maximum of 520 nm. This would likely interfere with the fluorescence of some dyes used in this study.

Response: Thanks for your valuable comments. Rhein has little effect on the dyes of EB, ATP, and $DiSC_3$ (5), but overlaps with the fluorescent dyes of ROS. However, the addition of a rhein solution group in the experimental setup removed the influence of rhein itself, and the experimental results were effective.

The docking results are very superficial. For example, there are no predicted binding energy data or images of the predicted docking sites.

Response: Thanks for your valuable comments. It has been added. His54, His58, Cys128, and Zn^{2+} serve as the active center. The binding energy of the docking is -6.3

kcal/mol and the docking image has been added. Line 369; Figure 4A

The authors need to double-check all references to make sure that it matches what they are claiming to cite.

Response: Thank you for your valuable feedback. We have carefully reviewed the references throughout the text and replaced any inappropriate ones.

Specific comments:

Line 22: Why is rhein a first-in-class inhibitor? There are many other plasmid conjugation inhibitors reported in the literature.

Response: Thanks for your valuable comments. The improper use of words caused confusion, and we have revised the wording. It was modified to "In this study, we identified rhein (RHE), a natural anthraquinone compound, as an effective inhibitor of IncFII-type plasmid transfer". Line 21-23

Line 37: The 'Importance' section reads like the abstract but just shortened. I think it needs to be re-written to indicate the significance of their research to a broad audience.

Response: Thanks for your valuable comments. We have rewritten the paragraph, specifically as follows: " Antimicrobial resistance in bacteria has become an increasingly severe global health challenge. The widespread dissemination of colistin resistance genes has markedly compromised the clinical efficacy of colistin, underscoring an urgent need for novel strategies to limit the spread of resistance determinants. In this study, we investigated the regulatory effects of rhein on IncFII-type plasmids and evaluated its intervention potential in the transmission of the colistin resistance gene mcr-1. Our results demonstrate that rhein effectively inhibits mcr-1 transfer in both in vitro and in vivo models, highlighting its potential as a promising therapeutic candidate for the prevention and control of antimicrobial

resistance gene dissemination.”　Line 38-46

Line 52: The use of 'ecosystem stability' is quite vague here. What do you mean?

Response: Thanks for your valuable comments. The original text intended to convey that antibiotic resistance genes (ARGs) can disrupt ecological balance and that they are a type of emerging environmental pollutant. Given the inappropriate expression of the relevant term, it has now been revised to“Antibiotic resistance genes (ARGs) have emerged as a global public health crisis, posing severe threats to clinical treatment efficacy and they are widely recognized as a category of emerging environmental pollutants”. Line 50-51

Line 57: This study (https://journals.asm.org/doi/full/10.1128/msphere.00170-23) should referenced here as it shows that growth in a biofilm promotes plasmid conjugation.

Response: Thanks for your valuable comments. It has already been cited.

Line 69: CTX-M-55 does not need to be italicised.

Response: Thanks for your valuable comments. It has been modified.

Line 92: What does 'anti-resistance agent' mean? I think it needs to be clarified.

Response: Thanks for your valuable comments. The original text stated that rhein, as an antimicrobial agent, has antibacterial effects on multi-drug resistant strains. It has been rewritten.　Line 90-100

Line 95: Is quorum-sing necessary for plasmid conjugation? Or does it just increase/promote transfer frequency?

Response: Thanks for your valuable comments. The original text contained an expression error, which has now been corrected “It is worth noting that existing studies have demonstrated that RHE disrupts bacterial membrane integrity and quorum sensing (QS) pathways, both of which play a critical facilitative role in

mediating bacterial plasmid conjugation(15-17)” Line 101-104

Line 114: What is rhein dissolved in?

Response: Thanks for your valuable comments. Rhein is dissolved in a $NaHCO_3$ solution.

Line 134: Overnight does not need to be capitalised.

Response: Thanks for your valuable comments. It has been modified.

Line 149: What does 'in triplicate' mean? Were these biological replicates? Was this on the same day or on different days?

Response: Thanks for your valuable comments. The improper use of words has caused you trouble, and it has been changed to "All experiments were performed with three biological replicates". This experiment includes three biological replicates, with three conjugation experiments repeated at different times. Line 157

Line 150: Should be 'In vivo conjugation assay'

Response: Thanks for your valuable comments. It has been modified. Line 158

Lien 160: What dose of avertin was used?

Response: Thanks for your valuable comments. It has been modified to “At 24 h post-infection, mice were anesthetized via intraperitoneal injection of 300 µL of 1.5% Avertin solution, followed by the collection of target organs”. Line 171-173

Line 167: Why was ONPG hydrolysis used to measure membrane permeability? I'm familiar with PI or NPN uptake being used to determine this in most studies.

Response: Thanks for your valuable comments. Rhein can cause fluorescence quenching of the probe, making it impossible to determine the permeability of the cell membrane through PI and NPN assays. Therefore, ONPG is used to concisely

measure cell membrane permeability.

Line 178-179: This should be changed to 'Ethidium bromide accumulation assay', as you are not directly measuring efflux, but rather accumulation of ethidium bromide. Also, in the Song et al. paper, I could not find the ethidium bromide accumulation assay method. Please double-check this reference and cite a more appropriate reference.

Response: Thanks for your valuable comments. It has been modified.

23. Paix O L, Rodrigues L, Couto I, et al. Fluorometric determination of ethidium bromide efflux kinetics in Escherichia coli. J BIOL ENG. 2009; 3: 18.

Line 202: Reference the Livak method here.

Response: Thanks for your valuable comments. It has been added.

Line 314: I could not find anything about bacterial conjugation in this citation (number 26).

Response: Thanks for your valuable comments. It has been modified.

20. Jia Y, Yang B, Shi J, et al. Melatonin prevents conjugative transfer of plasmid-mediated antibiotic resistance genes by disrupting proton motive force. PHARMACOL RES. 2022; 175: 105978.

30. Zhang S, Fang J, Liu H, et al. Enhanced plasmid-mediated conjugative transfer of resistance genes across bacterial species promoted by graphene oxide. ENVIRON SCI-NANO. 2024; 11: 831-45.

31. Piscon B, Esposito EP, Fichtman B, et al. The Effect of Outer Space and Other Environmental Cues on Bacterial Conjugation. MICROBIOL SPECTR. 2023; 11.

Line 325: This sentence needs a reference

Response: Thanks for your valuable comments. It has been added.

32. Li B, Srivastava S, Shaikh M, et al. Bioenergetic stress potentiates antimicrobial

resistance and persistence. NAT COMMUN. 2025; 16.

Line 361: Change 'confirmed' to 'suggested'

Response: Thanks for your valuable comments. It has been modified.

Line 382: csgA and csgB do not need to be capitalised.

Response: Thanks for your valuable comments. It has been modified.

Figure 7: Gene names should be italicised.

Response: Thanks for your valuable comments. It has been modified.

Line 442: Change 'potently inhibits' to 'reduces'

Response: Thanks for your valuable comments. It has been modified.

Line 445: Change 'cripples' to 'disrupts'

Response: Thanks for your valuable comments. It has been modified.

Line 485: This claim needs a reference to support it.

Response: Thanks for your valuable comments. It has been added.

45. Zhao R, Nawrocki A, M Ller-Jensen J, et al. Mechanistic divergence between SOS response activation and antibiotic-induced plasmid conjugation in Escherichia coli. MICROBIOL SPECTR. 2025; 13: e0009025.

Re: Spectrum03399-25R1 (**Rhein Reduces Conjugation of IncFII-Type Plasmids in *Escherichia coli* and Mitigates the Spread of Antibiotic Resistance Genes**)

Dear Prof. Zhongqiong Yin:

Thank you for the privilege of reviewing your work. Below you will find my comments, instructions from the Spectrum editorial office, and the reviewer comments.

Please return the manuscript within 60 days; if you cannot complete the modification within this time period, please contact me. If you do not wish to modify the manuscript and prefer to submit it to another journal, notify me immediately so that the manuscript may be formally withdrawn from consideration by Spectrum.

Revision Guidelines
To submit your modified manuscript, log into the submission site at https://spectrum.msubmit.net/cgi-bin/main.plex. Go to Author Tasks and click the appropriate manuscript title to begin. The information you entered when you first submitted the paper will be displayed; update this as necessary. Note the following requirements:

• Upload point-by-point responses to the issues raised by the reviewers in a file named "Response to Reviewers," NOT in your cover letter.
• Upload a compare copy of the manuscript (without figures) as a "Marked-Up Manuscript" file.
• Upload a clean .DOC/.DOCX version of the revised manuscript and remove the previous version.
• Each figure must be uploaded as a separate, editable, high-resolution file (TIFF or EPS preferred), and any multipanel figures must be assembled into one file.
• Any supplemental material intended for posting by ASM should be uploaded with their legends separate from the main manuscript. You can combine all supplemental material into one file (preferred) or split it into a maximum of 10 files with all associated legends included.

For complete guidelines on revision requirements, see our Submission and Review Process webpage. Submission of a paper that does not conform to guidelines may delay acceptance of your manuscript.

Data availability: ASM policy requires that data be available to the public upon online posting of the article, so please verify all links to sequence records, if present, and make sure that each number retrieves the full record of the data. If a new accession number is not linked or a link is broken, provide Spectrum production staff with the correct URL for the record. If the accession numbers for new data are not publicly accessible before the expected online posting of the article, publication may be delayed; please contact production staff (Spectrum@asmusa.org) immediately with the expected release date.

Publication Fees: For information on publication fees and which article types are subject to charges, visit our website. If your manuscript is accepted for publication and any fees apply, you will be contacted separately about payment during the production process; please follow the instructions in that e-mail. Arrangements for payment must be made before your article is published.

ASM Membership: Corresponding authors may join or renew ASM membership to obtain discounts on publication fees. Need to upgrade your membership level? Please contact Customer Service at Service@asmusa.org.

The ASM Journals program strives for constant improvement in our submission and publication process. Please tell us how we can improve your experience by taking this quick Author Survey.

Thank you for submitting your paper to Spectrum.

Sincerely,
Jian Peng
Editor
Microbiology Spectrum

Reviewer #2 (Comments for the Author):

Overall, the authors have addressed my concerns. I have minor grammatical and stylistic comments below:

Line 22: Again effective inhibitor is a strong claim. Change to 'In this study, we identified rhein (RHE), a natural anthraquinone compound, as an effective IncFII plasmid transfer reducing agent'.

Line 44: Change 'inhibits' to 'reduces'.
Line 85: Typo. 'linolec' should be 'linoleic acid'
Line 142: Overnight does not need to be capitalised
Line 194-195: This reads like instructions. It needs be changed to past tense.
Line 278: The use of 'prevent' here is grammatically incorrect and needs to be modified
Line 299: Was more than one type of IncFII plasmid tested?
Line 359: Typo. Should be 'proton motive force'
Figure 3: Indicate in the figure legend how the comparisons were made i.e. 40, 80, and 160 μg/mL was compared to control without rhein.
Figure 3: Some data (C, D and E) is shown as relative change whereas some is shown with raw data (A and B). I think it would be better to pick one for consistency.
Line 369: Should be past tense, change 'is' to 'was'.
Figure 5: The SEM images need a scale.
Line 393: What concentration of rhein treatment was used for the SEM images?
Line 404: If you are referring to genes, it should be italicised and not capitalised
Figure 6: IsrB should not be capitalised. Also, the x-axis needs to be labelled as 'Genes'. The y-axis has a typo, it should 'expression'
Figure 7C: finO and hok should not be capitalised
Line 530: Typo, change 'o' to 'to'
In the supplementary, the standard deviations for the conjugation frequencies should be added with a {plus minus} sign.

Reviewer #3 (Comments for the Author):

Comments:
1. Several sentences in the manuscript are unnecessarily lengthy and complex, which impairs readability. The authors are advised to revise these sentences to enhance clarity and conciseness, and to adopt more succinct expressions throughout the manuscript, parti cularly in the abstract and introduction sections.
2. Line 262: It is recommended that the specific determination criteria for the sub-inhibitory concentration be briefly supplemented in the Materials and Methods section.
3. Lines 358 and 392: The captions of Figure 3 and Figure 5 are overly concise. It is suggested that key explanatory information be added to these captions to better convey the core findings presented in the figures.
4. Line 434: Abbreviations such as Mpf and Dtr should be clearly defined at their first occurrence in the manuscript to ensure readability for all readers.
5. The discussion section could be improved by briefly comparing rhein with existing inhibitors of plasmid conjugation, which would help highlight the innovative advantages of rhein and strengthen the novelty of the study.
6. Lines 522-525: The conclusion section mentions a dual mechanism of rhein. It is recommended that the primary and secondary mechanisms be clearly differentiated, with a brief summary of their respective roles in inhibiting plasmid conjugation and blocking antibiotic resistance gene transmission.

Dear Editor and Reviewers,

We appreciate the opportunity to revise our manuscript titled " **Rhein Reduces Conjugation of IncFII-Type Plasmids in *Escherichia coli* and Mitigates the Spread of Antibiotic Resistance Genes" (Manuscript ID:Spectrum03399-25)** and are grateful for the insightful comments provided by the reviewers. Those comments are all valuable and very helpful for revising and improving our paper, as well as the important guiding significance to our researches. In the following, we have provided detailed responses to each of the reviewers' comments. Revised portion is highlighted in yellow the paper. Additionally, we have conducted a comprehensive revision of the entire manuscript. In this response letter, the reviewers' comments are presented in black, and our corresponding changes and additions to the manuscript are highlighted in blue text. We have tried our best to make all the revisions clear, and we hope that the revised manuscript meets the requirements for publication.

Reviewer #2 (Comments for the Author):

Overall, the authors have addressed my concerns. I have minor grammatical and stylistic comments below:

Line 22: Again effective inhibitor is a strong claim. Change to 'In this study, we identified rhein (RHE), a natural anthraquinone compound, as an effective IncFII plasmid transfer reducing agent'.

Response: Thanks for your valuable comments. It has been modified. Line 21-22

Line 44: Change 'inhibits' to 'reduces'.

Response: Thanks for your valuable comments. It has been modified. Line 43

Line 85: Typo. 'linolec' should be 'linoleic acid'

Response: Thanks for your valuable comments. It has been modified. Line 82

Line 142: Overnight does not need to be capitalised

Response: Thanks for your valuable comments. It has been modified. Line 141

Line 194-195: This reads like instructions. It needs be changed to past tense.

Response: Thanks for your valuable comments. It has been modified. "Ethidium bromide (EtBr) was added to a final concentration of 4 μg/mL, and the fluorescence intensity was measured after 5 min to assess the intracellular retention of EtBr ". Line 194-196

Line 278: The use of 'prevent' here is grammatically incorrect and needs to be modified

Response: Thanks for your valuable comments. It has been modified. "These results confirmed that RHE had no adverse impacts on the growth of donor and recipient strains at all tested concentrations, suggesting that its efficacy in reducing IncFII plasmid transfer was not mediated by the inhibition of bacterial proliferation". Line 276-279

Line 299: Was more than one type of IncFII plasmid tested?

Response: Thanks for your valuable comments. It has been modified. We tested a type of IncFII plasmid. We have corrected the sentence by changing the plural form of "plasmids" to the singular form. Line 299

Line 359: Typo. Should be 'proton motive force'

Response: Thanks for your valuable comments. It has been modified. Line 359

Figure 3: Indicate in the figure legend how the comparisons were made i.e. 40, 80, and 160 μg/mL was compared to control without rhein.

Response: Thanks for your valuable comments. It has been modified. Line 362-363

Figure 3: Some data (C, D and E) is shown as relative change whereas some is shown with raw data (A and B). I think it would be better to pick one for consistency.

Response: Thanks for your valuable comments. We have now revised Figure 3 to present all data as uniformly processed values, eliminating the inconsistency in presentation format.

Line 369: Should be past tense, change 'is' to 'was'.

Response: Thanks for your valuable comments. It has been modified. Line 370

Figure 5: The SEM images need a scale.

Response: Thanks for your valuable comments. It has been added.

Line 393: What concentration of rhein treatment was used for the SEM images?

Response: Thanks for your valuable comments. A rhein treatment concentration of 160 μg/mL was specified in the Materials and Methods section. Line 247

Line 404: If you are referring to genes, it should be italicised and not capitalised

Response: Thanks for your valuable comments. *csgA* and *csgB* refer to genes, and the text has been revised accordingly. Line 405-408

Figure 6: lsrB should not be capitalised. Also, the x-axis needs to be labelled as 'Genes'. The y-axis has a typo, it should 'expression'

Response: Thanks for your valuable comments. It has been modified.

Figure 7C: finO and hok should not be capitalised

Response: Thanks for your valuable comments. It has been modified.

Line 530: Typo, change 'o' to 'to'

Response: Thanks for your valuable comments. It has been modified. Line 540

In the supplementary, the standard deviations for the conjugation frequencies should be added with a {plus minus} sign.

Response: Thanks for your valuable comments. It has been modified.

Reviewer #3 (Comments for the Author):

Comments:

1. Several sentences in the manuscript are unnecessarily lengthy and complex, which impairs readability. The authors are advised to revise these sentences to enhance clarity and conciseness, and to adopt more succinct expressions throughout the manuscript, parti cularly in the abstract and introduction sections.

Response: Thanks for your valuable comments. We have simplified cumbersome and wordy sentences throughout the manuscript. For example, they are modified to " Novel therapeutic strategies independent of conventional antimicrobials are urgently needed. Line 20-21

Antibiotic resistance genes (ARGs) have become a serious global public health threat. They severely impair clinical treatment outcomes and are widely regarded as emerging environmental contaminants. Horizontal gene transfer (HGT) is a major driver, and plasmid-mediated conjugation is the most efficient route for ARG spread. Line 43-52

In such environments, conjugative plasmids facilitate efficient intercellular ARG transmission. Accordingly, conjugation serves as a core pathway for resistance spread, particularly for plasmid-carried resistance genes in clinical and high-density microbial niches(2, 3). Line 54-57

Blocking the conjugative horizontal transfer of ARGs is a promising and evolutionarily friendly intervention strategy. It can limit the transmission of resistance genes with minimal selective pressure on bacteria, thereby reducing the risk of new AMR generation. Line 72-75

However, the inherent toxicity of these compounds greatly limits their application as anti-plasmid agents. For instance, ethidium bromide has mutagenic effects, while cationic surfactants exhibit strong cytotoxicity to mammalian cells(9). Line 77-80

In addition to the above biological functions, accumulating evidence has confirmed

the broad-spectrum antibacterial activity of RHE against both Gram-positive and Gram-negative bacteria, including *Bacillus megaterium*, *Staphylococcus aureus* and *Cutibacterium acnes*(12). Line 92-95

In summary, sub-inhibitory RHE treatment (40-160 μg/mL) disrupted multiple bacterial physiological processes, including increased membrane permeability, decreased PMF and ATP production, altered ROS levels, and weakened efflux pump function. These combined damages collectively suppressed IncFII plasmid conjugation in *E. coli*. Line 352-356

This combined targeting mode is consistent with the development strategies of novel antibacterial agents. Such strategies take physicochemical damage (e.g., membrane disruption) as the core mechanism, accompanied by the regulation of biological pathways as auxiliary antibacterial approaches." Line 487-490

2. Line 262: It is recommended that the specific determination criteria for the sub-inhibitory concentration be briefly supplemented in the Materials and Methods section.

Response: Thanks for your valuable comments. It has been modified. "Our previous study confirmed that the minimum inhibitory concentration (MIC) of RHE against *E. coli* was 1280 μg/mL. To exclude the interference of RHE on bacterial growth, the growth curves of *E. coli* were determined at a series of subinhibitory concentrations". Line 130-133

3. Lines 358 and 392: The captions of Figure 3 and Figure 5 are overly concise. It is suggested that key explanatory information be added to these captions to better convey the core findings presented in the figures.

Response: Thanks for your valuable comments. It has been modified. "Figure 3. All data are presented as the mean ± SD of three independent biological replicates. Statistical significance was determined by one-way ANOVA. 40, 80, and 160 μg/mL was compared to control without rhein. *$P < 0.05$, **$P < 0.01$, ***$P < 0.001$, ****$P < 0.0001$." Line 360-363

"Figure 5. Rhein disrupts biofilm formation and cell morphology. (A) Scanning electron microscopy (SEM) images of bacterial cells, Scale bar=1μm , magnification ×15,000; (B) Quantification of biofilm formation in donor and recipient strains after treatment with 0, 40, 80, or 160 μg/mL RHE. Biofilm formation is presented as the mean ± SD of three biological replicates. Significant differences were evaluated by one-way ANOVA analysis and shown with *P<0.05, **P< 0.01, ***P< 0.001, ****P< 0.0001." Line 393-398

4. Line 434: Abbreviations such as Mpf and Dtr should be clearly defined at their first occurrence in the manuscript to ensure readability for all readers.

Response: Thanks for your valuable comments. It has been modified. Line 433

5. The discussion section could be improved by briefly comparing rhein with existing inhibitors of plasmid conjugation, which would help highlight the innovative advantages of rhein and strengthen the novelty of the study.

Response: Thanks for your valuable comments. It has been added. "Several conjugation inhibitors have been reported, including unsaturated fatty acids (2-HDA)(46), nucleoside analogs (AZT)(47), synthetic small molecules, and natural products such as baicalin and dihydroartemisinin(33, 48). Most of these agents exert their effects through a single mechanism, for instance, targeting the TrwD ATPase, interfering with QS, or disrupting the type IV secretion system(33, 46, 48). In comparison, RHE exerts a unique dual-mode action. It damages bacterial membrane structure, reduces energy metabolism, and blocks the LuxS/AI-2 QS pathway, thereby effectively inhibiting IncFII plasmid conjugation. Furthermore, RHE significantly reduces *mcr-1* transmission both *in vitro* and *in vivo*, whereas most documented inhibitors lack confirmed *in vivo* activity. With its multi-target mode of action, low tendency to induce resistance, and natural source, RHE exhibits distinct advantages over most currently available conjugation inhibitors and serves as a promising candidate for curbing the dissemination of antibiotic resistance. " Line 505-517

6. Lines 522-525: The conclusion section mentions a dual mechanism of rhein. It is recommended that the primary and secondary mechanisms be clearly differentiated, with a brief summary of their respective roles in inhibiting plasmid conjugation and blocking antibiotic resistance gene transmission.

Response: Thanks for your valuable comments. It has been modified. "Mechanistically, this inhibition occurs through sequential disruption of key processes supporting horizontal gene transfer: first, impairment of membrane integrity and proton motive force; second, reduction of intracellular ATP levels; and third, suppression of quorum sensing and biofilm formation. Collectively, these multi-modal effects act synergistically to limit plasmid dissemination. " Line 531-534

Re: Spectrum03399-25R2 (**Rhein Reduces Conjugation of IncFII-Type Plasmids in *Escherichia coli* and Mitigates the Spread of Antibiotic Resistance Genes**)

Dear Prof. yin zhongqiong:

Your manuscript has been accepted, and I am forwarding it to the ASM production staff for publication. Your paper will first be checked to make sure all elements meet the technical requirements. ASM staff will contact you if anything needs to be revised before copyediting and production can begin. Otherwise, you will be notified when your proofs are ready to be viewed.

Data Availability: ASM policy requires that data be available to the public upon online posting of the article, so please verify all links to sequence records, if present, and make sure that each number retrieves the full record of the data. If a new accession number is not linked or a link is broken, provide production staff with the correct URL for the record. If the accession numbers for new data are not publicly accessible before the expected online posting of the article, publication may be delayed; please contact ASM production staff immediately with the expected release date.

Publication Fees: For information on publication fees and which article types have charges, please visit our website. We have partnered with Copyright Clearance Center (CCC) to collect author charges. If fees apply to your paper, you will receive a message from no-reply@copyright.com with further instructions. For questions related to paying charges through RightsLink, please contact CCC at ASM_Support@copyright.com or toll free at +1-877-622-5543. CCC makes every attempt to respond to all emails within 24 hours.

ASM Membership: Corresponding authors may join or renew ASM membership to obtain discounts on publication fees. Need to upgrade your membership level? Please contact Customer Service at Service@asmusa.org.

Embargo Policy: A press release may be issued as soon as the manuscript is posted on the Spectrum Latest Articles webpage. The corresponding author will receive an email with the subject line "ASM Journals Author Services Notification" when the article is available online.

The ASM Journals program strives for constant improvement in our submission and publication process. Please tell us how we can improve your experience by taking this quick Author Survey.

Thank you for submitting your paper to Spectrum.

Sincerely,
Jian Peng
Editor
Microbiology Spectrum

Reviewer #2 (Comments for the Author):

The authors have addressed all of my concerns. This is an interesting study that will be valuable to AMR and natural product research.

Reviewer #3 (Comments for the Author):

The author has resolved all my concerns.
