# Supplementary material for: Rhein reduces conjugation of IncFII-type plasmids in Escherichia coli and mitigates the spread of antibiotic resistance genes
Source: Microbiol Spectr. 2026 Jul 6;14(8):e03399-25. doi: 10.1128/spectrum.03399-25 (PMC13435978; doi:10.1128/spectrum.03399-25)
Supplement: Supplementary file 2 [file spectrum.03399-25-s0001.docx]

**Supplemental materials**

**Table S1 The primer sequences of PCR**

| Gene | Primer (5’-3’) | Product (bp) |
| --- | --- | --- |
| *mcr-1* | F:GCTCGGTCAGTCCGTTTGTTCTTG | 1110 |
|  | R:GAGCATACCGACATCGCGGCA |  |
| *bla*_CTX-M-55_ | F:ACGCTGTTGTTAGGAAGTGTGC | 659 |
|  | R:TTATCCCCCACAACCCAGGAAG |  |

**Table S2 The primer sequences of RT-qPCR**

| Gene | Primer (5’-3’) |
| --- | --- |
| 16S rRNA | F:CGGTGAATACGTTCYCGG |
|  | R:GGWTACCTTGTTACGACTT |
| *finO* | F:TGGCGTGGTGACATTGATGA |
|  | R:GACACTGAAGCGGAAAACGG |
| *hok* | F:TACTTCCGTGCTTCGTAGGC |
|  | R:GTGTGCCACGGTATTGTGTT |
| *relE* | F:CCGTCTGACATCCCTGGTTT |
|  | R:CTGCAGCCAAAACGGTGTAG |
| *traC* | F:TCCACTTCCAGAGCCGTTTC |
|  | R:CTCCGGTCAGTCTTCTGTCGT |
| *traF* | F:GCACTCATGGTGAAAAGCCC |
|  | R:GCCATTCTGTATCCCGGTGT |
| *traR* | F:ATTCAACGCACAACGTCACA |
|  | R:TGAAGCATGCGGAAATCCTG |
| *trbB* | F:GGAAAGGCGGTATCCCCCTG |
|  | R:TACTGCCACCAGTTTGACCC |
| *trbF* | F:TCGCACATTGGGGACAGAAA |
|  | R:ATGGCCGACCCGAAATTACT |
| *luxS* | F:ACTTACCAGATGCACTCGTTG |
|  | R:AGTGCCAGTTCTTCGTTGCT |
| *lsrK* | F:AACTTAAAGAACTGCACAACA |
|  | R:ACTTAAAGCCAGTGTTTGTCC |
| *lsrB* | F:TCTGATACTAAACCGGAGTGC |
|  | R:TGGCTTTGTCTTTATTCACCT |
| *mtn* | F:TGCCACAATTTCAACGTCCC |
|  | R:CAGCCAGGAACTCATCGAAG |
| *fimE* | F:ACGTCGTTATCTTACCG |
|  | R:AATCCGTTCTTCAGTCG |
| *fimB* | F:ATATTATCTCGACTTCCGGTG |
|  | R:TAAGTCGCGTATCTATTCCC |
| *rpoS* | F:ACATCCTGGCCGAT |
|  | R:AACAGCCATTTGACGAT |
| *csgA* | F:GTAATAATAGCGGCCCAA |
|  | R:TTACCGCCGCCATGCTG |
| *csgB* | F:CTTATGGTAATACTGCGATG |
|  | R:GAATAGCCATTTGCGACT |

Figure S1. Growth curves of rhein on donor bacteria (A) and recipient bacteria (B) at sub-inhibitory concentration.

**Table S3 The MICs of wild strain and transconjugants**

| Drugs  Strains | **MICs（μg/mL）** | | | |
| --- | --- | --- | --- | --- |
|  | COL | NaN₃ | TET | AMP |
| J53 | <1 | 512 | <1 | <1 |
| J-DH5a(*bla*_CTX-M-55_ *positive*) | <1 | 512 | <1 | 128 |
| J0514-1(*mcr-1* positive) | 16 | 256 | 2 | 8 |
| J0514-2(*mcr-1* positive) | 16 | 256 | 32 | 128 |
| RCAD 0514 | 16 | 16 | 64 | >512 |
| DH5a(*bla*_CTX-M-55_ *positive*) | <1 | 8 | <1 | >512 |

Note: COL, colistin; NaN₃, Sodium Azide; TET, tetracycline; AMP, Ampicillin

Figure S2 Agarose gel electrophoresis of *bla*_CTX-M-55_ (A) and *mcr-1* (B) gene detection PCR product.

Figure S3 Transfer frequency of *E. coli* carrying IncFII plasmid after supplementing with exogenous AI-2.

Table S4 The frequency of conjugation transfer between *Escherichia coli* DH5α carrying IncFII and *Escherichia coli* J53

| RHE concentration (μg/mL) | Conjugation frequency  （Mean±SD） | |
| --- | --- | --- |
| 0 | | 1.81×10^-5^±0.74 |
| 40 | | 9.37×10^-6^±2.15 |
| 80 | | 7.70×10^-6^ ±1.98 |
| 160 | | 6.40×10^-6^±1.33 |

Table S5 The frequency of conjugation transfer between *Escherichia coli* DH5α carrying IncFII and *Escherichia coli* J53 at different temperatures

| RHE concentration (μg/mL) | Conjugation frequency at different temperatures  （Mean±SD） | | |
| --- | --- | --- | --- |
|  | 30℃ | 37℃ | 42℃ |
| 0 | 9.18×10^-6^±1.22 | 4.06×10^-5^±1.09 | 9.30×10^-5^±1.20 |
| 40 | 6.89×10^-6^±0.45 | 3.52×10^-5^±1.41 | 7.21×10^-5^±0.29 |
| 80 | 7.94×10^-6^±0.46 | 1.99×10^-5^±0.21 | 7.93×10^-5^±0.57 |
| 160 | 2.39×10^-6^±0.46 | 1.99×10^-5^±0.20 | 6.97×10^-5^±0.46 |

Table S6 The frequency of conjugation transfer between *Escherichia coli* DH5α carrying IncFII and *Escherichia coli* J53 at different times

| RHE concentration (μg/mL) | Conjugation frequency at different times  （Mean±SD） | | | |
| --- | --- | --- | --- | --- |
|  | 6 h | 12 h | 24 h | 48 h |
| 0 | 1.30×10^-4^±0.2 | 4.75×10^-5^±1.15 | 1.34×10^-4^±0.44 | 1.18×10^-4^±0.38 |
| 40 | 6.59×10^-5^±1.04 | 2.45×10^-5^±0.41 | 7.45×10^-5^±0.84 | 8.03×10^-5^±0.36 |
| 80 | 6.74×10^-5^±1.06 | 2.83×10^-5^±0.26 | 6.6×10^-5^±1.39 | 7.94×10^-5^±0.75 |
| 160 | 2.02×10^-5^±0.59 | 1.24×10^-5^±0.29 | 6.28×10^-5^±0.57 | 6.84×10^-5^±0.36 |

Table S7 The frequency of conjugation transfer between *Escherichia coli* RCAD 0514 (*mcr-1 positive* ) and *Escherichia coli* J53

| RHE concentration  s (μg/mL) | Conjugation frequency  （Mean±SD） |
| --- | --- |
| 0 | 5.07×10^-4^±0.74 |
| 40 | 8.22×10^-5^±1.36 |
| 80 | 6.35×10^-5^±1.12 |
| 160 | 4.52×10^-5^±1.43 |
